# Exploring alternative approaches to precision medicine through genomics and artificial intelligence – a systematic review

**DOI:** 10.3389/fmed.2023.1227168

**Published:** 2023-10-02

**Authors:** Hassan Mumtaz, Muhammad Saqib, Sidra Jabeen, Muhammad Muneeb, Wajiha Mughal, Hassan Sohail, Myra Safdar, Qasim Mehmood, Muhammad Ahsan Khan, Syed Muhammad Ismail

**Affiliations:** ^1^Maroof International Hospital, Islamabad, Pakistan; ^2^Khyber Medical College, Peshawar, Pakistan; ^3^Liaquat National Hospital, Karachi, Pakistan; ^4^Department of Medicine, Dow University of Health Sciences, Karachi, Pakistan; ^5^Armed Forces Institute of Cardiology and National Institute of Heart Diseases (AFIC-NIHD), Rawalpindi, Pakistan; ^6^Department of Medicine, King Edward Medical University, Lahore, Pakistan

**Keywords:** artificial intelligence, genomics, biomarkers, precision medicine, humans

## Abstract

The core idea behind precision medicine is to pinpoint the subpopulations that differ from one another in terms of disease risk, drug responsiveness, and treatment outcomes due to differences in biology and other traits. Biomarkers are found through genomic sequencing. Multi-dimensional clinical and biological data are created using these biomarkers. Better analytic methods are needed for these multidimensional data, which can be accomplished by using artificial intelligence (AI). An updated review of 80 latest original publications is presented on four main fronts—preventive medicine, medication development, treatment outcomes, and diagnostic medicine—All these studies effectively illustrated the significance of AI in precision medicine. Artificial intelligence (AI) has revolutionized precision medicine by swiftly analyzing vast amounts of data to provide tailored treatments and predictive diagnostics. Through machine learning algorithms and high-resolution imaging, AI assists in precise diagnoses and early disease detection. AI’s ability to decode complex biological factors aids in identifying novel therapeutic targets, allowing personalized interventions and optimizing treatment outcomes. Furthermore, AI accelerates drug discovery by navigating chemical structures and predicting drug-target interactions, expediting the development of life-saving medications. With its unrivaled capacity to comprehend and interpret data, AI stands as an invaluable tool in the pursuit of enhanced patient care and improved health outcomes. It’s evident that AI can open a new horizon for precision medicine by translating complex data into actionable information. To get better results in this regard and to fully exploit the great potential of AI, further research is required on this pressing subject.

## Introduction

Today’s health care has a growing emphasis on personalized treatments. Precision medicine, often known as personalized medicine, is a data-driven approach that seeks to enhance clinical results by individually configuring treatments for each patient, given a patient’s state (consisting of covariate history, demographics, genetic makeup, diagnostic test findings, etc.). For example, type 2 diabetic patients are usually started on the drug metformin (1), however different people may react differently to the same treatment. Scientists are now beginning to comprehend that genetics might contribute to the variability in response to drug treatment, explaining why every patient responds to treatment differently (2). With precision medicine, such knowledge of genetic changes aids clinicians in selecting a treatment strategy that is ideal for each patient in terms of their well-being and desired outcome. It also presents the chance to activate other fields of research that more effectively target the disease (3).

The study of developing computational technologies that, like people, are capable of performing tasks like sensing, learning, reasoning, and taking action led to the development of the field of artificial intelligence (AI), which was first recognized in the 1960s by researchers in the engineering and cognitive sciences. Early AI systems mainly depended on guidelines created by experts to mimic how humans would approach these activities. As research on numerical techniques incorporating ideas from computers, optimization, and statistics to automatically “train” programs for executing certain tasks by processing data began, a subfield of artificial intelligence (AI) known as machine learning (ML) has also evolved.

Artificial intelligence has made significant strides in the field of medicine. For instance, Esteva et al. (4) and Hekler et al. (5) employed clinical imaging data to create classification models to help doctors diagnose skin cancer, skin lesions, and psoriasis in the field of visually focused specialties like dermatology (6, 7). A deep convolutional neural network (DCNN) model was specifically trained by Esteva et al. (4) utilizing 129,450 pictures to categorize images as either keratinocyte carcinoma or seborrheic keratosis; and malignant melanoma or benign nevus.

Using examples from recent research, we hope to present a clear, understandable, and technologically accurate picture of machine learning (ML), which is frequently referred to as artificial intelligence (AI) in the medical literature, as it exists today and what it can do for health and medicine.

## Methodology

### Study design

To provide the best available scientific evidence related to our topic, we conducted a systematic review (SR) of original articles.

### Search strategy

This review was conducted in accordance with the Preferred Reporting Items for systematic reviews and meta-analysis (PRISMA) and is reported according to the PRISMA statement 2020. Relevant articles were obtained by searching: PubMed; Google Scholar, and PLOS One. In all these databases, we applied filters to reliably identify original articles and searched these databases from 2011 to 16th November 2021. Keywords for the investigation were identified using the contributing authors’ knowledge and we used Boolean search: (Artificial Intelligence OR Machine Learning OR Deep learning) AND (Precision Medicine). Searches were supplemented by hand searching and retrieval of any additional articles fulfilling the inclusion criteria that were cited in the reference list.

### Inclusion criteria

Published studies fulfilling the following criteria were included: (1) Study design: original articles were included only. (2) Only those articles were included which were published between 2011 to 16th May 2023. (3) Only those studies were included that successfully highlighted the role of AI in precision medicine on four major grounds, i.e., preventive medicine, drug development, treatment outcome, and diagnostic medicine.

### Exclusion criteria

All those studies were excluded: (1) study design: Knowledge, attitude, and prevalence (KAP) or cross-sectional studies. (2) Studies that were unpublished or non-peer-reviewed. (3) Studies that were close-access. (4) Full text was available in languages other than English. (5) Studies that highlighted the role of AI in precision medicine in domains other than preventive medicine, drug development, treatment outcomes, and diagnostic medicine.

### Data extraction

**Figure 1:**
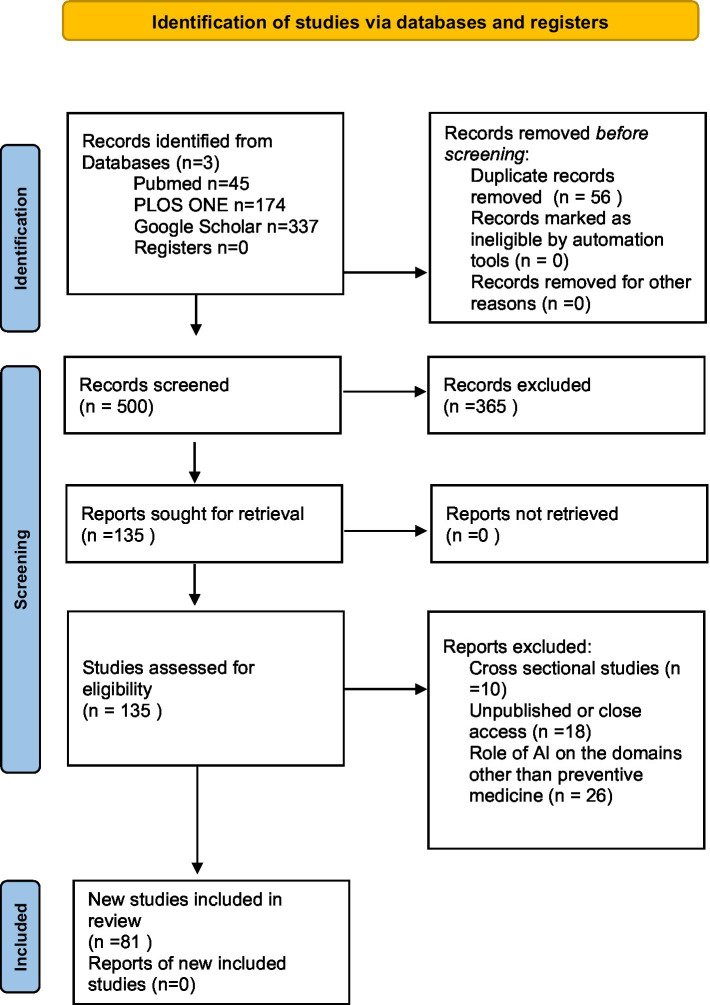
PRISMA flowchart (8).

The screening of the studies was conducted by four independent reviewers to assess whether the studies would be satisfied according to inclusion criteria after reading titles, abstracts, and full texts. H. Arksey and L. O’Malley’s methodological framework was used for the inclusion of selected studies according to the eligibility criteria. After study selection, data was extracted and documented on an Excel sheet. Grey literature was excluded and duplicated articles were also removed. Extracted articles were visually tabulated using the PRISMA flow diagram 2020 as shown in Figure 1.

## Results

We included more than 81 studies in our review under the following four headings and have described notable studies in the text. The studies are described further in Table 1 and in the discussion section of the review.

**Table 1:** Studies extracted.

#	Study name	Location	No. of patients	AI algorithm	Application
	Preventive medicine				
1	Wu et al. (9)	Taiwan	56	Random forest	Characterization of TMAO productivity from carnitine challenge for the facilitation of personalized nutrition and microbiome signatures discover
2	Abedi et al. (10)	Tennessee and greece	428	Artificial neural network (ANN)	Novel screening tool for the recognition of ACI and differentiation of ACI from stroke mimics at the initial examination.
3	Brennan et al. (11)	Florida, USA	150	MySurgeryRisk algorithm	To assess the usability and accuracy of the MySurgeryRisk algorithm for preoperative risk assessment using a simulated workflow for the real-time, intelligent, decision support platform.
4	Marshall et al. (13)	Australia and New Zealand	178	Neuro-fuzzy inference system (DENFIS)	Evolving connectionist system (ECOS) versus algebraic formulas for prediction of renal function from serum creatinine
5	Mencattini et al. (14)	France	102	Support vector machine (SVM), dynamic feature selection, multiple linear regression	A precision approach aimed at classifying samples of speech produced by children with developmental disorders (DD) and typically developing (TD) children.
6	Steele et al. (1)	England	80,000	Cox models, random forests, and elastic net regression	Prognostic modeling from data in electronic health records (EHR) using machine-learning approaches in coronary artery disease
7	Romero-Rosales (15)	Mexico	5,220	Genetic algorithms, LASSO, and step-wise	Use of machine learning models in Improving Alzheimer’s disease prediction
8	Saha (16)	Australia	77	MRI-based deep learning CNN model	Application of deep learning in predicting motor outcomes in preterm infants from early brain MRI
9	Lu et al. (17)	Scotland	25	Bayesian hierarchical vector autoregressive (VAR) model	Prediction of patient health outcomes using Bayesian hierarchical vector autoregressive model
10	Popov et al. (18)	Russia	Dataset consisted of 392 disease-associated and 154 benign point mutations located in 64 transmembrane proteins	BorodaTM	Prediction of disease-associated mutations in the transmembrane regions of proteins with known 3D structure.
11	Parikh (6)	USA	110,000	(Density-based clustering) technique	A machine learning approach to identify distinct subgroups of veterans at risk for hospitalization or death.
12	Weng et al. (2)	UK	378,256	Random forest, logistic regression, gradient boosting machines, neural networks	Can machine learning improve cardiovascular risk prediction using routine clinical data?
13	Stuckey et al. (3)	USA	512	The cPSTA System	Cardiac phase space tomography: a novel method of assessing coronary artery disease utilizing machine learning
14	Karpati (12)	Israel	60,423	“traj” R package, “NbClust” algorithm (R package), and K-means clustering	Patient clusters based on HbA1c trajectories: a step toward individualized medicine in type 2 diabetes
15	Zhao et al. (19)	USA	1,693	Support Vector Machine (SVM)	Exploration of machine learning techniques in predicting multiple sclerosis disease course
16	Baydoun et al. (4)	Lebanon	30	MATLAB-based tool and algorithm	High precision digitization of paper-based ECG records: a step toward machine learning
17	Kim et al. (7)	Korea	24,269	Random forest classifiers, support vector machines, gradient-boosted decision trees (XGBoost), and artificial neural networks	Predicting participation in cancer screening programs with machine learning
18	Blasco et al. (20)	Europe	512	Biosigner algorithm	A pharmacometabolomics approach in a clinical trial of ALS: Identification of predictive markers of progression
	Drug development				
1	Ott et al. (21)	USA	10	Various algorithms (not distinctly mentioned)	An immunogenic personal neoantigen vaccine for patients with melanoma
2	Li et al. (22)	USA	The cancer cell line encyclopedia (CCLE) 400 cell lines.	Mixture regression model-based method	Drug sensitivity prediction with high-dimensional mixture regression.
3	Miranda et al. (23)	Brazil	987 cell lines in the genomics of drug sensitivity in cancer database	Five classification algorithms and four regression algorithms representing diverse methodologies, including tree-, probability-, kernel-, ensemble-, and distance-based approaches	Predicting drug sensitivity of cancer cells based on DNA methylation levels
4	Fang et al. (24)	China	CCLE dataset.	Quantile regression forest	A quantile regression forest-based method to predict drug response and assess prediction reliability
5	Vitali et al. (25)	Italy	Genes from 104 cases of primary TNBC	Boolean Networks (BNs)	A network-based data integration approach to support drug repurposing and multi-target therapies in triple negative breast cancer
6	Tang et al. (26)	USA	seqFISH+ data of mouse cortex and the NanoString CosMx Spatial Molecular Imager (SMI) data of non-small cell lung cancer samples	spaCI	Deciphering spatial cellular communications through adaptive graph model
	Treatment outcome				
1	Bartlett et al. (27)	USA	184	ComBat	Pretreatment and early-treatment cortical thickness are associated with SSRI treatment response in major depressive disorder
2	Albizu et al. (28)	USA	14	Support vector machine (SVM) learning algorithm	Machine learning and individual variability in electric field characteristics predict tDCS treatment response
3	Nguyen et al. (29)	USA	Crossover trial	Crossover generalized outcome weighted learning	Estimating individualized treatment regimes from crossover designs
4	Rajpurkar et al. (30)	USA	518	Transparent reporting of a multivariable prediction model	Evaluation of a machine learning model based on pretreatment symptoms and electroencephalographic features to predict outcomes of antidepressant treatment in adults with depression
5	Tomalin et al. (31)	USA	266	Seven different machine-learning methods were selected from the R suite “caret” (glmnet, nnet, pls, pam, rf, svmLinear)	Early quantification of systemic inflammatory proteins predicts long-term treatment response to Tofacitinib and Etanercept
6	Webb et al. (32)	USA	216	Machine learning with a Personalized Advantage Index (PAI)	Personalized prediction of antidepressant v. placebo response: evidence from the EMBARC study
7	Kazemipoor et al. (33)	Malaysia	70	Adaptive neuro-fuzzy inference (ANFIS) method.	Appraisal of adaptive neuro-fuzzy computing technique for estimating anti-obesity properties of a medicinal plant
8	Yoon et al. (34)	South Korea	89 training +60 test sets	Penalized logistic regression, penalized discriminant analysis, and various other algorithms	Use of radiomics to predict the efficacy of immunotherapy by deciphering tumor microenvironment
9	Bradley et al. (35)	United Kingdom	77 studies	Bayesian network	Prediction of prognostic outcomes after pancreatic adenocarcinoma resection using AI
10	Huang et al. (36)	USA	60 human cancer cell lines (NCI-60)	Support vector machine (SVM) algorithm	Prediction of response to cancer drug therapy using open-source machine learning algorithm
11	Lind et.al (37)	USA	Genomics of Drug Sensitivity in Cancer (GDSC) project	Random forest classification models	Prediction of drug activity against cancerous cells using machine learning
12	Kaissis et al. (38)	Germany	55	Gradient-boosted-tree algorithm	A machine learning algorithm predicts molecular subtypes in pancreatic ductal adenocarcinoma with differential response to gemcitabine-based versus FOLFIRINOX chemotherapy
13	Kleinerman et al. (39)	USA	10,000	Differential Prototypes Neural Network (DPNN	Application of Novel Deep Machine Learning model in the selection of depression treatment
14	Mirchi et al. (40)	Canada	28	Perceptron	The virtual operative assistant; artificial intelligence tool for simulation-based training in surgery and medicine
15	Christie et al. (41)	USA	1,494	SuperLearner	Dynamic multi-outcome prediction after injury.
16	Ramella et al. (42)	Italy	91	Ensemble learning method	A radiomic approach for adaptive radiotherapy in NSCLC
17	Sharma et al. (43)	India	–	1. Multi-task deep neural network 2. Multi-task kernel learning 3. Hierarchical Bayesian model	Intelligent chatbot for prediction and management of stress.
18	Sheikhalishahi et al. (44)	Italy	73,000	BiLSTM model and 1-layer artificial neural network	Benchmarking machine learning models on multi-center eICU critical care dataset.
19	McCoy et al. (45)	USA	3,634	Natural language processing	Differences among Research Domain Criteria score trajectories by Diagnostic and Statistical Manual categorical diagnosis during inpatient hospitalization
20	Jiang et al. (46)	USA	6,726	Causal modeling with internal layers (CAMIL), and treatment feature interactions (TFI)	A clinical decision support system learned from data to personalize treatment recommendations for preventing breast cancer metastasis
21	Lin et al. (47)	USA	35,334	Recurrent neural networks (RNN) with long short-term memory (LSTM)	Analysis and prediction of unplanned intensive care unit readmission using recurrent neural networks with long short-term memory
22	Merali et al. (48)	Canada	757	Random forest, support vector machine, logistic regression, simple decision tree, and artificial neural network (ANN) model	Using a machine learning approach to predict outcomes after surgery for a +degenerative cervical myelopathy
23	Jiang et al. (49)	USA	SKMEL-2 and HS294T cell lines	Generalized linear model (GLM)	Network assessment of demethylation treatment in melanoma: differential transcriptome-methylome and antigen profile signatures
24	Panda (50)	India	–	Endo microrobots, geminoids, nanobots	Use of robots in dentistry: a fact or a fiction
	Diagnostic medicine				
1	Kline et al. (51)	USA	60	Automated deep learning approach using a convolutional neural network	Automatic semantic segmentation of kidney cysts in MR images of patients affected by autosomal-dominant polycystic kidney disease
2	Wang et al. (52)	China	240	Region-based convolutional neural network	Evaluation of rectal cancer circumferential resection margin using faster region-based convolutional neural network in high-resolution magnetic resonance images
3	Gates et al. (53)	Texas, USA	23	Random forest,13 support vector machines, and neural network classifiers	Imaging-based algorithm for the local grading of glioma
4	Wang et al. (54)	China	539	Radiomic nomogram	Intratumoral and peritumoral radiomics analysis for preoperative Lauren classification in gastric cancer
5	Nielson et al. (55)	USA	586	Topological data analysis (TDA)	Uncovering precision phenotype-biomarker associations in traumatic brain injury using topological data analysis
6	Ko et al. (56)	Taiwan	1742	Supervised machine learning (SML)	Clinically validated machine learning algorithm for detecting residual diseases with multicolor flow cytometry analysis in acute myeloid leukemia and myelodysplastic syndrome
7	Gastel et al. (57)	Netherland and USA	440 abdominal magnetic resonance images	Automated segmentation artificial deep neural network	Automatic measurement of kidney and liver volumes from MR images of patients affected by autosomal dominant polycystic kidney disease
8	Zhang et al. (58)	China	215	Convolutional neural network constructed based on Single Shot MultiBox Detector (SSD)	Real-time gastric polyp detection using convolutional neural networks
9	Lim et al. (59)	Korea	69	Deep learning-based semantic segmentation network.	Reproducibility of automated habenula segmentation *via* deep learning in major depressive disorder and normal controls with 7 Tesla MRI
10	Mokhtari et al. (60)	USA	66	Dynamic brain networks	Dynamic fMRI networks predict success in a behavioral weight loss program among older adults
11	Lyra et al. (61)	Germany, Australia	6	Deep learning-based algorithm	A deep learning-based camera approach for vital sign monitoring using thermography images for ICU patients
12	de Jong et al. (62)	Netherland	285 in a single center; 223 patients included in a multicenter	CT-based radiomics	Applicability of a prognostic CT-based radiomic signature model trained on Stage I-III non-small cell lung cancer in stage IV non-small cell lung cancer
13	Dekhil et al. (63)	USA	283	Automated autism diagnosis system	Using resting-state functional MRI to build a personalized autism diagnosis system
14	Badgujar et al. (64)	India	UniProt database and the NCBI database	Computational analysis using *in silico* tools	Computational analysis of high-risk SNPs in human CHK2 gene responsible for hereditary breast cancer: a functional and structural impact
15	Bahado-Singh et al. (65)	USA	24 late-onset AD (LOAD) and 24 cognitively healthy subjects.	Artificial Intelligence (AI) methodologies including Deep Learning (DL) followed by Ingenuity Pathway Analysis (IPA)	Artificial intelligence and leukocyte Epigenomics: evaluation and prediction of late-onset Alzheimer’s disease
16	Kim et al. (66)	Korea	Test dataset (100 cases) and another dataset (399 cases).	Four machine learning algorithms: C5.0, random forest (RF), support vector machine (SVM), and k-nearest neighbor (KNN).	Development of machine learning models for diagnosis of glaucoma
17	Lynch et al. (67)	USA	358	LASSO machine-learning	The effect of neighborhood social environment on prostate cancer development in black and white men at high risk for prostate cancer
18	Keek et al. (68)	Netherland	444	Cox proportional hazards regression and random survival forest (RSF).	Computed tomography-derived radiomic signature of head and neck squamous cell carcinoma (peri)tumoral tissue for the prediction of locoregional recurrence and distant metastasis after concurrent chemoradiotherapy
19	Rieg et al. (69)	Germany	10,646	White-box machine learning	Demonstration of the potential of white-box machine learning approaches to gain insights from cardiovascular disease electrocardiograms
20	Gaudelet et al. (70)	UK, Spain, France	4,788	Neural network-based data–integration framework	Unveiling new disease, pathway, and gene associations via multi-scale neural network
21	Maggio et al. (71)	Italy	498	CDRP (Concatenated Diagnostic-Relapse Prognostic) architecture	Distillation of the clinical algorithm improves prognosis by multi-task deep learning in high-risk neuroblastoma
22	Takahashi et al. (72)	Japan	177	The deep neural network (DNN)	Automated system for diagnosing endometrial cancer by adopting deep-learning technology in hysteroscopy
23	Jiang et al. (73)	China	7,909 microscopic images	Convolutional neural networks with small SE-ResNet module	Breast cancer histopathological image classification using convolutional neural networks with a small SE-ResNet module
24	Szpiech et al. (74)	USA	Component analysis of 29 cancers	Non-negative matrix factorization and principal component analysis	Prominent features of the amino acid mutation landscape in cancer
25	Kundu et al. (75)	India, South Korea	5,856 chest X-ray images	Convolutional neural network models: GoogLeNet, ResNet-18, and DenseNet-121.	Pneumonia detection in chest X-ray images using an ensemble of deep learning models
26	Araújo et al. (76)	Brazil	84	Support vector machine (SVM) algorithm	Finding reduced Raman spectroscopy fingerprint of skin samples for melanoma diagnosis through machine learning
27	Goetz et al. (77)	USA	Five first-year medical students and three fourth-year medical students were recruited, along with four first-year engineering graduate students and three fourth-year com- puter/data science graduate students.	–	Perceptions of virtual primary care physicians: a focus group study of medical and data science graduate students
28	Guijo-Rubio et al. (78)	Spain	United Network for Organ Sharing database	Multilayer Perceptron (MLP), Random Forest (RF), Gradient Boosting (GB) or Support Vector Machines (SVM), among others.	Statistical methods versus machine learning techniques for donor-recipient matching in liver transplantation
29	Schaack et al. (79)	Germany	371	Machine-learning-based solutions like decision tree (DT), random forest (RF), support vector machine (SVM), and deep-learning neural networks (DNNs).	Comparison of machine-learning methodologies for accurate diagnosis of sepsis using microarray gene expression data
30	Gennatas et al. (80)	USA	235	Logistic regression (generalized linear model, GLM), classification, and regression trees (CART), logistic regression with elastic net regularization (GLMNET), support vector machines (SVM) with a radial basis kernel, MediBoost Tree-Structured Boosting, random forest (RF) and gradient boosting machine (GBM)	Preoperative and postoperative prediction of long-term meningioma outcomes
31	Maulucci et al. (81)	Italy	26	Decision-Support-System (DSS)	Phase separation of the plasma membrane in human red blood cells as a potential tool for diagnosis and progression monitoring of type 1 diabetes mellitus
32	Singh et al. (82)	USA	10,936	Random forests (the Weill Cornell model).	Comparing a novel machine learning method to the Friedewald formula and Martin-Hopkins equation for low-density lipoprotein estimation
33	Dai et al. (83)	Taiwan	649	Convolutional neural networks	Assessing the severity of positive valence symptoms in initial psychiatric evaluation records: Should we use convolutional neural networks?

### Preventive medicine

In the field of preventive medicine, several studies were identified that utilized AI algorithms for various applications. Wu et al. (9) conducted a study in Taiwan with 56 patients, employing a random forest algorithm to characterize TMAO productivity from carnitine challenge for personalized nutrition and microbiome signature discovery (9). Another method to predict glomerular filtration rate (GFR) was developed by Marshall et al. (13) using evolving connectionist systems (ECOS), which are cutting-edge computing structures that can be trained to produce precise results from a given set of input variables. Abedi et al. (10) developed a novel screening tool using an Artificial Neural Network (ANN) to differentiate acute cerebral infarction from stroke mimics (10). In order to classify speech samples produced by children with developmental disorders (DD) and normally developing (TD) children, Mencattini et al. (14) investigated the potential application of a precision approach to the construction of a statistical learning algorithm. Brennan et al. (11) evaluated the usability and accuracy of the MySurgeryRisk algorithm for preoperative risk assessment. These studies demonstrate the potential of AI algorithms in improving risk assessment and personalized screening tools in preventive medicine.

### Drug development

Several studies focused on drug development utilized AI algorithms for predicting drug sensitivity and identifying therapeutic targets. Ott et al. (21) used various algorithms to develop an immunogenic personal neoantigen vaccine for melanoma patients (21). A unique adaptive graph model incorporating mechanisms of attention called spaCI was proposed by Tang et al. (26) to decode the cell-to-cell contacts from SCST profiles. The active ligand-receptor (L-R) signaling axis across adjoining cells is identified by spaCI, which takes into account both the geographical placements of cells and their gene expression profiles. SpaCI has outperformed currently known approaches in comparison tests on both simulation data and actual SCST datasets.

In Triple Negative Breast Cancer, which has a little-known biology and lacks defined molecular targets, Vitali et al. (25) used network-based modelling to support repurposing of drugs and multi-target treatments. Li et al. (22) employed a mixture regression model-based method to predict drug sensitivity using the Cancer Cell Line Encyclopedia (CCLE) dataset (22). Sofia P. For the purpose of forecasting drugs reactions, Fang et al. (24) developed a three-step quantile regression forest (QRF) technique and applied it to the Cell Line Encyclopaedia (CCLE) dataset. Miranda et al. (23) used diverse classification and regression algorithms to predict drug sensitivity based on DNA methylation levels (23). These studies highlight the role of AI algorithms in advancing drug development by facilitating personalized treatment strategies and predicting drug response.

### Treatment outcome

In the domain of treatment outcomes, numerous studies utilized AI algorithms to predict treatment responses and assess patient outcomes. Bartlett et al. (27) employed the ComBat algorithm to associate cortical thickness with selective serotonin reuptake inhibitor treatment response in major depressive disorder (27). Using information from a 2×2 crossover research, Nguyen et al. (29) offered a method for calculating the ideal individualized treatment regime (ITR). Albizu et al. (28) used a Support Vector Machine (SVM) algorithm to predict transcranial direct current stimulation (tDCS) treatment response based on electric field characteristics (28). With the goal of developing predictive models, Tomalin et al. (31) calculated longitudinal profiles for 92 inflammatory and 65 cardiovascular-related proteins from the blood of psoriasis patients at baseline and 4 weeks after receiving tofacitinib or etanercept treatment. Following tofacitinib or etanercept treatment, they were able to correctly predict the 12-week clinical endpoint for psoriasis, revealing a robust predictive protein signature that included well-known psoriasis markers like IL-17A and IL-17C, highlighting the potential for biologically significant response predictions using blood protein data. These studies demonstrate the potential of AI algorithms in predicting treatment outcomes and personalizing therapeutic interventions.

### Diagnostic medicine

Several studies focused on diagnostic medicine and utilized AI algorithms for automated analysis and classification. Kline et al. (51) developed an automated deep-learning approach using a convolutional neural network to segment kidney cysts in MR images of patients with autosomal-dominant polycystic kidney disease (51). Wang et al. (52) evaluated rectal cancer resection margins using a region-based convolutional neural network (52). E.D.H. Gates et al. (53) utilized random forest, support vector machines, and neural network classifiers to develop an imaging-based algorithm for the local grading of glioma (53). Topological data analysis (TDA), a machine-learning method, was used by Nielson et al. (55) in traumatic brain injury (TBI) patients to discover data-driven patterns in patient outcomes and identify potential indicators of recovery. These studies demonstrate the potential of AI algorithms in improving diagnostic accuracy and automating image analysis in various medical conditions.

## Discussion

Our findings revealed literature elaborating on the use of artificial intelligence in various aspects of precision medicine, upgrading the clinical tools in diagnostic and preventive medicine, drug development, and treatment outcomes.

### Preventive medicine

In conducting this review, we looked at the literature that elaborated on the use of machine learning and artificial intelligence techniques in preventative medicine.

#### In cardiovascular diseases

An English study described the use of machine learning models to forecast patient mortality in coronary artery disease using information taken from electronic medical records. Over 82,000 patients’ data were included in the study. Without any prior data processing, machine learning techniques surpassed traditional models in prognosis prediction. Elastic net Cox regression yielded a C-index of 0.801 as opposed to a conventional Cox model’s 0.793 (1). Many individuals who would benefit from preventative care cannot be identified using current methods for predicting cardiovascular risk, while others undergo needless intervention. By taking advantage of the complex relationships between risk factors, machine learning gives the chance to increase accuracy. In order to compare four machine learning algorithms with the established CVD risk algorithm advised by the American Heart Association/American College of Cardiology (ACC/AHA), Weng et al. (2) used 378,256 healthy patients. Their prospective cohort study found that neural networks had the highest predictive value. According to the study’s findings, machine learning considerably increases the accuracy of cardiovascular risk prediction, allowing doctors to identify more patients who could benefit from preventive care while avoiding the needless treatment of others (2).

Cardiac Phase Space Tomography Analysis (cPSTA), which uses elastic net method-based machine-learned linear models, analyses thoracic phase signals to find specific mathematical and tomographic features associated with the presence of flow-limiting CAD without any radiation risk, according to a study done to compare the diagnostic accuracy of cPSTA and coronary angiography in patients with chest pain (3).

Heart disease diagnosis often relies heavily on electrocardiograms (ECGs). However, the majority of illness patterns are based on outdated information and inaccurate stepwise algorithms. In order to improve the detection of advanced T-wave shape and spatial QRS-T angle, a study investigated the application of advanced machine learning in the form of a MATLAB-based tool and algorithm that converts a printed or scanned format of the ECG into a digital ECG signal. In the investigation, 30 ECG-scanned curves were used. When the results were validated using signals from various records, each of which had a 1,000-data-point interval and contained at least four heartbeats, they showed very high correlation values for several common ECG parameters, including PR intervals of 0.984 +/−0.021, QRS intervals of 1+/− SD, QT intervals of 0.981 +/− 0.023, and RR intervals of 1 +/− 0.001. The study found that existing paper or scanned ECGs can yield digital ECG signals with greater than 95% accuracy. This enables the use of historical ECG signals in machine learning algorithms to recognize heart disease trends and assist in the diagnosis and prognostic assessment of individuals with cardiovascular disease (4).

#### In allergic medicine

A study published in the United States reported the implementation of machine learning models in the identification of food allergy (FA) biomarkers and possible epigenetic targets for the disease using DNA methylation data achieved perfect classification accuracy on completely hidden test cohorts by using subset analysis of 18-featured potential CpG biomarkers. This was done to address the worrying limitation of laboratory tests not being able to distinguish between people who have FA and those who are merely sensitized to foods. The excellent accuracy on a huge number of hidden data permutations, where the samples in the training, cross-validation, and hidden sets were repeatedly randomly assigned, served as additional proof of the effectiveness of these machine learning classifiers and the 18 CpGs. Seven of the 13 genes were previously linked to FA, and many of the FA-discriminating genes discovered in this study were highly related to the immune system (5).

#### In public health hospital management

Population health management systems have placed a strong emphasis on identifying those who are at high risk for imminent hospitalization or mortality. Due to the inclusion of a limited number of variables in the prior literature, the previous studies faced a lot of heterogenicity in generalizing high-risk people. Care Assessment Needs (CAN) score is a commonly used VA model that predicts a patient’s percentile risk of hospitalization or death at 1 year, and it was recently utilized in cross-sectional research to identify high-risk Veterans. Patient-level data was broken down into 119 unique variables, including sociodemographic characteristics, comorbidities, medications, vitals, labs, and prior utilization. This was the largest study to date that used ML clustering approaches to divide a population into subgroups based on their potential for harm (6). In another investigation, researchers in South Korea utilized a variety of machine learning models to foretell how many people will show up for cancer screenings. These included RF classifiers, SVM, gradient-boosted decision trees, and artificial neural networks (ANN). Using stratified cluster sampling, a total of 24,269 people (10,611 homes) took part in the survey. Area under the Receiver Operating Characteristics (AUC-ROC), Average Precision (Area under the Precision-Recall curve), and Accuracy were selected as the three measures of precision. Nonetheless, there was a little uptick in participation from 50.1% in 2015 to 55.6% in 2019. The paper’s primary flaw was that it relied on self-reported data from the Korea National Health and Nutrition Examination Survey (7). In a pilot study, the efficacy of a My Surgery Risk algorithm to predict postoperative complications was more accurate (AUCs ranged from 0.64 to 0.85) as compared to the initial physicians’ risk assessments (AUCs ranged between 0.47 and 0.69) with greater AUCs for predicted absolute risks for all complications (*p* < 0.002 each) except cardiovascular. This real-time intelligent technology projected postoperative problems with the same or higher accuracy as our sample of physicians (11). AI has made several fascinating advances recently, one of which is a virtual doctor. A group of future doctors and data scientists thought this idea was brilliant since it would save money and would be very accurate. However, participants were hesitant to share mental health issues and indicated worries about data privacy and responsibility in the event of a misdiagnosis while interacting with virtual artificial intelligence physicians (77). By analyzing a patient’s previous occurrences, the Bayesian Hierarchical Vector Autoregressive Model (VAR) was able to forecast the patient’s medical and psychological states with greater accuracy than any other VAR model (17).

#### In diabetes mellitus

To account for the diversity within the community of persons with type 2 diabetes, new recommendations from the American Diabetes Association and the European Association for the Study of Diabetes advocate developing customized objectives. Current recommendations do not address how to include glycemic level trajectories when describing individual risk, despite evidence suggesting that glycemic fluctuation over time is a significant independent risk factor for death. Karpati et al. (12) used unsupervised machine learning on longitudinal HbA1c trajectories to identify clusters of patients with distinct risks for diabetes-related complications, suggesting that these clusters can serve as the foundation for developing individualized models to personalize glycemic targets. Using a random forest model, they were able to reliably reconstruct the three distinct distributions of HbA1c trajectories among 60,423 patients: stable (*n* = 45,679), falling (6,084), and rising (8,660) trends. Results from the clinical relevance analysis showed a J-shaped connection between HbA1c levels and the risk for outcomes (12).

#### In neuroscience and neurodevelopmental disorders

A study looked at the efficacy of machine learning in estimating Multiple Sclerosis (MS) progression and found that the popular linear Support Vector Machine (SVM) technique performed just slightly better than the gold standard of logistic regression (LR), with an accuracy rate of 64% vs. 62%. When initial MRI data, such as T2 lesion volume and BPF, were incorporated, the total accuracy increased to 68% for LR and 70% for SVM (19). The use of a machine learning algorithm using Genome-wide association studies (GWAS) for early detection of Alzheimer’s disease revealed that the LASSO model was most effective in predicting Alzheimer’s disease out of the three learning models while another study showed the use of ANN to be a diagnostic tool for the recognition of Acute Cerebral Ischemia (ACI) and distinguishing it from stroke cases in the Emergency department with an average sensitivity and specificity of ANN for the diagnosis of ACI based on the 10-fold cross-validation analysis of 80.0% (95% confidence interval, 71.8–86.3) and 86.2% (95% confidence interval, 78.7–91.4), respectively (10, 15). A multidrug clinical trial to demonstrate the use of a pharmacometabolomic approach in predicting the progression of Amyotrophic lateral sclerosis (ALS) revealed that multidrug treatment modifies different metabolic pathways and metabolic features provided by these analyses regarding the specific mechanism of action of these drugs has effects for the development of other drugs in ALS and other neurodegenerative disorders (20). Assuming that acoustic features of vocal production cannot be efficiently used as a direct marker of Developmental Disorders (DD), a study from 2018 looked into the possibility of applying a precision strategy for the development of a statistical learning algorithm with the goal of classifying samples of speech produced by children with DD and typically developing (TD) children (14).

#### In microbiology and genetics

The use of AI in the field of preventative genomics was demonstrated in another study. Using machine learning, researchers were able to develop a technique called BorodaTM (Boosted Regression trees for Disease-Associated mutations in Trans Membrane proteins) that can distinguish between pathogenic and nonpathogenic point mutations in the Transmembrane regions of proteins with known 3D structure (18).

Human health can be significantly impacted by the ability of gut microbiota to create atherogenic trimethylamine N-oxide (TMAO) from carnitine by decomposing meals and medicines containing this compound. Two obligate anaerobes, E. timonensis, and I. massiliensis, were discovered as possible significant actors for converting carnitine to TMA in the human gut using an oral carnitine challenge test (OCCT) utilized as a method to detect gut microbial signatures and promote individualized carnitine ingestion (9).

#### In nephrology

The study demonstrates that the performance of evolving connectionist systems (ECOS) for GFR prediction may be enhanced somewhat by extra regression studies before clinical usage in various populations and that ECOS are more accurate than Algebraic formulas in ordinary clinical practice. In order to facilitate future prospective multicenter research, we have created a Web-based version of GFRDENFIS; it is hoped that the resulting computational models will help researchers better understand the biological mechanisms that regulate renal function and provide new strategies for preventing or treating renal disease. Results showed that machine intelligence might be implemented with more precision than such algebraic formulae (13).

#### In oncology

A Prognostic Bayesian network was utilized to provide personalized pre-operative predictions of post-resection 12-month survival rates and post-operative prognosis updates in patients with pancreatic ductal cancer; 77 papers were incorporated in the creation of the network. Accepting up to four missing data points in the sample resulted in an AUC of 0.7 (*p* value: 0.001; 95% CI 0.589–0.801) for pre-operative forecasts. When tested on a dataset with up to 6 missing pre-operative data points and 0 missing post-operative data points, an AUC of 0.8 (*p* value: 0.000; 95% CI:0.710–0.870) was attained for post-operative prognostic updating. In the case when up to two data points were absent from the post-operative validation dataset, the AUC was reduced to 0.7 (*p* = 0.000; 95% CI:0.667–0.818). A tool like this has the potential to be extremely useful in improving the quality of collaborative decision-making in clinical practice, and with further development, it may even provide a means of delivering individualized precision medicine (35).

### Drug development

Based on our review of the available literature, we learned that AI has already been put to use in the cancer medication discovery process. For Triple Negative Breast Cancer, the most effective multi-target medicines were identified by *in vitro* research that examined the use of network-based modeling inside a unique bioinformatics pipeline to incorporate data from diverse sources (25).

Predictive biomarkers can identify patients who have been affected by treatments and are a major driving force behind the creation of potent medicines. The average treatment impact in the general population is given importance in traditional randomized clinical trials (RCTs), therefore this conventional technique requires personalization and re-designs, as there is insufficient scientific support for the assumption that treatment efficacy correlates with homogeneity in the targeted group. Before beginning phase III trials, there must be a promising candidate signature, which adds a great deal of complexity. To deal with this issue, Freidlin and Simon (84) suggested the adaptive signature design (ASD), which involves dividing patients into training and validation sets for the development and confirmation of a predictive classifier in a single (pivotal) experiment. The MAGE-A3 immunotherapeutic effectiveness has recently been tested in patients with stage IIIB or IIIC melanoma in the adjuvant context, with disease-free survival being the goal (85).

For Triple Negative Breast Cancer, the most effective multi-target medicines were identified by *in vitro* research that examined the use of network-based modeling inside a unique bioinformatics pipeline to incorporate data from diverse sources (25).

Another study demonstrated the creation of a neoantigen vaccination with the capacity to not only increase the size of preexisting neoantigen-specific T cell populations but also to generate a wider repertoire of new T cell characteristics in patients with cancer. Out of a total of 97 neoantigens, vaccine-induced polyfunctional CD4+ and CD8+ T cells targeted 60 and 16%, respectively (21).

We found three studies demonstrating the use of artificial intelligence methods for drug sensitivity and response, which are an essential part of precision medicine. One of them demonstrated how to apply a mixture regression to assess the population heterogeneity and feature selection for each of the subpopulations, both of which are crucial in drug sensitivity prediction. The model was estimated utilizing the imputation-conditional consistency method, and encouraging findings showed that the mixture regression model significantly improved upon its predecessors in terms of predicting drug sensitivity and feature selection (22). In the second article, the Cell Line Encyclopaedia (CCLE) dataset was modeled using genomic parameters such as baseline gene expressions, mutation status, and copy number variations, and a three-step quantile regression forest (QRF) technique was presented to predict drug responses. It improved upon previously known methods for identifying medication responses. The method achieved a better accuracy level for detecting drug response as compared to the already available tools. The approach not only gave a good ‘point’ prediction but also provided an interval prediction of the drug response (24). In the third study, researchers evaluated the cytotoxic effects of eight different anti-cancer medications by analyzing the DNA methylation patterns of 987 different cell lines included in the Genomics of Drug Sensitivity in Cancer (GDSC) database. They compared five different categorization algorithms and four different regression strategies. The feasibility of identifying clinical responses for human tumors based on models developed from cell lines was assessed using data from the Cancer Genome Atlas. In general, the algorithms were unable to determine any patterns that accurately predicted the outcomes for the patients. However, the major drawbacks of this study were that the researcher only concentrated on DNA methylation profiles in isolation and did not include other types of molecular features which could have also likely modified treatment responses. Secondly, the treatment-response data was mis-proportioned, meaning that not all response classes included an equal number of patients. Therefore, the next research might investigate how class differences affect how well a model performs (23).

### Treatment outcome

Treatment responses and outcomes based on individualized differences are the main focus of precision medicine. We extracted 24 articles elaborating on the use of AI techniques for improving treatment outcomes in precision medicine.

The use of AI in neuropsychiatric precision medicine and their treatment outcomes were evaluated in seven articles. The first study showed the use of Establishing Moderators and Biosignatures of Antidepressant Response in Clinical Care (EMBARC) to inspect pre-treatment and early treatment changes in brain structure occurring within the first week of selective serotonin reuptake inhibitor (SSRI) and placebo treatment in patients with major depressive disorder (MDD) and found that bilaterally, rostral ACC cortical thickness (CT) alterations in the first week of treatment were associated with the eventual change in symptom severity during a trial of sertraline, while RMF pre-treatment CT, and CT and volume alterations in the first week of treatment were associated with the change in symptom severity during the placebo trial (27). The second study was a randomized controlled trial that compared cognitive therapy (CT) and interpersonal psychotherapy (IPT) for the treatment of depression by using the Personalized Advantage Index (PAI) to predict results for patients. Identified a minor difference (2.7 points on the BDI-II) in mean depression severity between patients advised to get CT and those recommended to receive IPT when PAI was present; however, this difference was observed only in patients recommended to receive CT (86). There was another trial study that compared the effectiveness of 8 weeks of sertraline versus placebo for adults suffering from depression. This study used machine learning in conjunction with a PAI index to create individualized treatment plans for patients suffering from major depressive episodes on the basis of endophenotype profiles mixed with clinical and demographic factors discovered that older patients and those with smaller impairments in cognitive control displayed better results to SSRI (32). The fourth study involved the use of machine learning models constructed *via* using classification-regression trees (CRT) and support vector machines (SVM) and GWAS data to evaluate duloxetine (SSRI) response and outcomes in patients with MDD showed that the models were characterized by a favorable sensitivity but specificity remained satisfactory at best (87).

Evidence from the fifth study demonstrated the use of machine learning in the form of gradient-boosted decision trees (GBDTs) to accurately determine acute improvement in individual depressive symptoms with antidepressants based on pre-treatment depression symptom scores and electroencephalography (EEG) revealed prejudiced presentation for identifying improved performance in specific symptoms reflected in high C index scores of 0.8 or higher on 12 of 21 clinician-rated symptoms (30). The sixth article showed Super-learning (SL) as a methodology to combine all the prediction algorithms and produce a final model for predicting the treatment outcome of Substance Use Disorder (SUD) using data from 99,013 SUD treatment patients. The area under the receiver operating characteristics curve (AUC) for all the algorithms was between 0.793 and 0.820, while SL was found to be superior to all but one of the algorithms. The study also aimed to introduce SL methodology to analysts and practitioners (88). The seventh article proposed the development of an AI application “Chatbot” using neural network and machine learning techniques to train the data in the application, guess the most accurate level of stress, and allow it to ask questions from the user/patient and using the answers to estimate the stress level and its management individualized for every user/patient (43). All these studies denote that AI models and algorithms can be used as a way forward in the treatment of depression, stress, and other neuropsychiatric disorders.

Predicting the pattern of readmission to the Intensive Care Unit (ICU) and creating an effective discharge decision-making support system for physicians and ICU experts were both featured in an article as examples of machine learning approaches. A key factor in ICU readmissions was found to be the LSTM-based solution’s capacity to identify significant volatility and instability among ICU patients. Also, compared to conventional models, the AUC for ML models predicting ICU readmissions was 0.791 (95% CI, 0.782–0.800), and their specificity was 0.742 (95% CI, 0.718–0.766) (47) while another study proposed a public benchmark suite to address four areas of critical care, namely mortality prediction, estimation of length of stay, patient phenotyping and risk of decompensation and compared the performance of both clinical models as well as baseline and deep learning models using electronic Intensive Care Unit (eICU) dataset of around 73,000 patients (44). A study used a natural language processing tool to obtain estimates of 5 Research Domain Criteria (RDoC) domains from the admission note and discharge summary, and then used linear regression to determine the change in each symptom domain during admission, finding that worsening of symptoms was the least common outcome in negative symptoms (0.4%) while greatest in cognitive symptoms (25.8%). The study was conducted on a retrospective cohort derived from an inpatient psychiatry unit at a large teaching hospital (45).

An essential aim of Precision medicine is the creation of optimal and individualized treatment regimens and results in cancer therapy, and four papers addressed this topic. In the first piece, we saw how a machine learning technique called Support Vector Machine (SVM) may be used in conjunction with a more traditional recursive feature elimination (RFE) method to predict a patient’s unique reaction to a medicine based on their gene expression profiles. The National Cancer Institute panel’s information on gene expression and medication response was utilized to develop patient-specific predictive models. A wide range of cancer cell lines had their responses to drugs accurately predicted by the models (36). Another study looked at how well machine learning algorithms could identify pancreatic adenocarcinoma molecular subtypes using radiomic characteristics, how well each subtype would respond to gemcitabine vs. FOLFIRINOX chemotherapy, and how long patients would live after treatment ended. With the use of immunochemical staining for the marker KRT81, subtypes of pancreatic ductal carcinoma were established in a total of 55 individuals. Machine learning was used to train 70% of the patient data in order to predict the remaining patient subtypes. Sensitivity, specificity, and ROC-AUC were all found to be statistically significant at 0.90 STDEV 0.7, 0.92 STDEV 0.11, and 0.93 STDEV 0.07. Even though the KRT81 subtype’s patients fared better in response to gemcitabine-based chemotherapy, their median overall survival was lower (38). A CDSS learned from data that recommends the optimal treatment decisions based on a patient’s features to prevent breast cancer metastasis was proposed in another article (DPAC: Clinical Decision Support System for Making Personalized Assessments and Recommendations Concerning Breast Cancer Patients). The analysis used a 5-fold cross-validation analysis to evaluate the chances of patients who followed DPAC’s advice to become metastasis-free within 5 years with those who did not. Yet DPAC advised that many node-negative individuals’ risks of metastasis were raised by treatment. This debate underlined the need to finalize DPAC in order to provide the highest quality patient-specific therapy recommendations (46). In 2018, researchers in the United States set out to confirm the widespread impact of epigenetic treatment, learn more about the role played by melanoma’s metastatic states, and assess the effectiveness of epigenetic therapy in melanoma cells of varying metastatic potential at the systems level. The results showed that treatment with DAC methylation with 5-Aza-2′-deoxycytidine of melanoma cells strongly affected early melanoma advancement by reactivating many genes and can be useful to halt cancer at pre-metastatic stages (49).

Machine learning algorithms in the form of novel methods like finite element models (FEM) and SVM algorithms were able to classify transcranial direct current stimulation (tDCS) treatment responders and non-responders with 86% accuracy based on patterns of current characteristics. The study provided the first evidence that pattern recognition analyses of MRI-derived tDCS current models can provide individual prognostic classification of tDCS treatment response with significant accuracy (28). Blood levels of 92 inflammatory and 65 cardiovascular disease proteins in psoriasis patients were measured before and after 4 weeks of therapy with Etanercept or tofacitnib using machine learning techniques such as bagging and ensembles. According to the canonical effectiveness goal for psoriasis, PASI75, patients were classified as responders if their PASI dropped by 75% or more after 12 weeks of therapy, and as non-responders otherwise. While other blood classifiers were knocked by simple models trained using Psoriasis Area Severity Index scores, the W0 classifier for tofacitnib greatly exceeded the predictions generated using merely the PASI score and indicated some potential advantage in a clinical scenario (31).

The application of an Adaptive neuro-fuzzy Inference System (ANFIS) technique to estimate the anti-obesity properties of a medicinal plant using SPSS analysis showed While the body weight, body fat percentage, and body mass index (BMI) of individuals in the CE group decreased significantly relative to the placebo group, there were still drawbacks to the models, such as the fact that they are quite demanding in terms of calculation time (33). An ML algorithm called Super Learner was used to evaluate precision medicine in trauma and to uncover patient-specific modifiable variables crucial to the trajectory of the patient for various major outcomes following severe trauma in 1494 critically injured patients. This study concluded that ML algorithms can help to transform data of trauma patients into real-time, dynamic decision-making support based on the algorithm’s excellent cross-validation prognostication of death, multi-organ failure, transfusion across multiple post-injury time points, and good prediction of acute respiratory distress syndrome and venous thromboembolism (41).

After applying ML approaches to the problem of predicting the outcomes of patients undergoing degenerative cervical myelopathy (DCM), a *post hoc* study showed that the RF and SVM models were superior to the LR, DT, and ANN models. The model found that pre-operative disease severity, duration of DCM symptoms, age, BMI, and smoking status were all predictive of worse surgical outcomes (48). Positive outcomes have been observed from using AI-enabled dental robots. Patients’ CT scans are used by robotic implant systems such as Dental Nanobots, the Geminoids family of robots, Endo Microbots, and Yomi, which have been approved by the FDA. Bone surface milling, hole drilling, deep saw osteotomy cuts, plate selection for osteosynthesis, and preoperative planning for orthognathic surgery have all benefited from robotic technology for some time (50).

## Diagnostic medicine

### In diagnostic oncology

Sixteen papers were chosen to provide context for AI and ML’s role in diagnostic oncology.

#### Gliomas

Estimating the local glioma grade according to the WHO grading system by using a multiclass machine learning model trained on preoperative image data and spatially specific tumor samples revealed that random forest was the best algorithm tested and clinical imaging data can predict pathological grading of the tumor with higher accuracy (53).

#### Gastric cancer

The use of radiomics in preoperative prognostic prediction in diagnostic oncology was demonstrated in four articles. First of them used a radiomic nomogram for preoperative prediction of the Lauren classification in gastric cancer (GC) by combining intratumoral, peritumoral, and partial clinical information and revealed that it incorporated the combined radiomic signature, age, CT T stage, and CT N stage and outperformed the other models with a training AUC of 0.745 and a validation AUC of 0.758. The nomogram showed promising results in distinguishing the Lauren diffuse type from the intestinal type, which is necessary for a practical therapeutic approach (54).

#### Non-small cell lung cancer (NSCLC)

The other three articles studied the use of radiomic features on non-small cell lung cancer (NSCLC). The first one aimed to validate the use of a CT-based radiomic signature in prognostic value of stage IV NSCLC patients and concluded that the signature does have satisfactory performance in predicting prognosis but not as high as for stage I-III patients, while the second study used radiomics to extract valuable features from diagnostic imaging to characterize tumour pathology of NSCLC and to guide personalized treatment in a study on 91 stage III NSCLC patients and 230 textural features extracted from the CT images using an ensemble learning method to classify the data into adaptive or non-adaptive during chemo radiotherapy on the basis of starting CT stimulation and showed promising results (AUC 0.82), while in the third article, Yoon et al. (34) explored radiologic phenotyping using a radiomics approach to assess the immune microenvironment in NSCLC using Single-sample gene set enrichment analysis on two independent NSCLC cohorts (training dataset comprised 89 NSCLCs and the test set included 60 cases of lung squamous cell carcinoma and adenocarcinoma) with the final model on the test set having an AUC of 0.684, it was concluded that radiomics approach can be used to interrogate an entire tumour in a noninvasive manner and provide added diagnostic value to identify the immune microenvironment of NSCLC, in particular, Th2 cell signatures (34, 42, 62).

#### Squamous cell carcinoma

In contrast to the aforementioned statement, a study that used Cox proportional hazards regression and random survival forest (RSF) as ML methods to evaluate the use of radiomic features derived from contrast-enhanced CT images to predict overall survival (OS), locoregional recurrence (LRR) and distant metastases in stage III and IV head and neck squamous cell cancer (HNSCC) patients treated with chemo radiotherapy found that the peritumoral radiomics based prediction models performed poorly in predicting OS, LRR, and DM (68).

#### Leukemias

Another article demonstrated the application of SML and AI approach to develop an algorithm by combining GMMbased phenotype representation with support vector machine (SVM) supervised model trained on a huge number of Multicolor flow cytometry (MFC) data can rapidly classify specimens with a high accuracy rate of detecting Minimal residual disease (MRD) post-chemotherapy in patients of acute myeloid leukemia (AML) and Myelodysplastic syndrome (MDS) (56).

#### Rectal cancer

Automatic image recognition took only 0.2 s after introducing a faster region-based convolutional neural network trained on high-resolution MRI to diagnose any circumferential resection margin in rectal cancer. This algorithm had a higher accuracy of 0.932, sensitivity of 0.838, and specificity of 0.956 (52).

#### Breast cancer

The CHK2 protein is a tumor-suppressor gene with a high vulnerability risk of developing hereditary breast cancer, and researchers in India used computational analysis to uncover nsSNPs which are harmful to the structure and/or function of this protein. SIFT, Align GVGD, SNAP-2, PROVEAN, Poly-Phen-2, PANTHER, PhD-SNP, MUpro, iPTREE-STAB, Consurf, InterPro, NCBI Conserved Domain Search tool, ModPred, SPARKS-X, RAMPAGE, Verify-3D, FT Site, COACH, and PyMol were used for the analysis. Seven of the 78 projected functionally most important SNPs in the human CHK2 gene. Researchers also found that changing the serine to phenylalanine at codon 415 represents a significant alteration in the native CHK2 protein, which might lead to its dysfunction and, ultimately, cancer (64).

Breast carcinoma histopathology images were automatically classified into benign, malignant, and 8 subtypes using an improved convoluted neural network consisting of a convolutional layer, a small SE-ResNet module, and a fully connected layer, which achieved the same performance with fewer parameters than the older models. Accuracy ranged from 98.87 to 99.34% for the binary classification, and from 90.67 to 99.34% for the multiclass classification. The study also utilized the Gauss error scheduler, a unique learning rate scheduler that removes the need for the user to manually adjust the learning rate parameter for the Stochastic Gradient Descent (SGD) method, thus improving the decision-making ability of radiologists (73).

#### Prostate cancer

The impact of Neighbourhood socioeconomic (nSES) factors in precision medicine for prostate cancer development was studied by calculating model-based nSES exposure scores and revealed that the 5-year predicted probability of prostate cancer was greater in men with a high nSES score and noses a later time to diagnosis (67).

#### Neuroblastoma

To enhance prognostic prediction in high-risk patients with neuroblastoma, Maggio et al. (71) presented a clinical approach called Concatenated Diagnostic-Relapse Prognostic (CDRP) for multi-task deep learning. The interpretability analysis of the model demonstrated that the SEQC-NB data may be naturally ordered for illness severity on a manifold and that this new feature space can be defined by using only one layer of the CDRP-N. While SEQC-NB training yields no discernible benefit, CDRP increases Matthews Correlation Coefficient (MCC) in validation for the Overall Survival (OS) endpoint and is the first model to improve on the High-Risk cohort, to the best of our knowledge (EFSHR, OSHR). Cross-validation on TGt for the HR tasks confirms the superiority of the CDRP-N and CDRP-A + CDRP-N architectures, with CDRP-N embedding linked to increased severity. These results were achieved using the TARGET-NB dataset (71).

#### Endometrial tumors

Implementation of a deep neural network (DNN)-based automated system using deep earning method to evaluate the presence of endometrial tumors from hysteroscopy images was studied on 177 patients with hysteroscopy history and classified into 5 groups- those with a normal endometrium, uterine myoma, endometrial polyp, atypical endometrial hyperplasia (AEH), and endometrial cancer, respectively. The results revealed that the diagnostic accuracy for endometrial cancer was 80, 89, and 90% using the traditional method, continuity analysis, and combined three neural networks, respectively (72).

#### Skin cancer

By analyzing the well-known “Raman biological fingerprint region” (800–1800 cm-1), we were able to train a machine learning model to use Raman skin human tissue spectra in distinguishing between malignant Melanoma (ME) and benign Melanocytic Nevus (MN) with a high level of accuracy (AUC 0.98, 95% CI 0.97–0.99). Importantly, they used a miniaturized spectral range (896–1,039 cm-1) to create a high-performance model (AUC 0.97, 95% CI 0.95–0.98), demonstrating that only a single and reduced fragment of the biological fingerprint Raman region was necessary to differentiate benign versus malignant skin lesions, thereby paving the way for a much more exclusive Raman spectrometer for a faster cancer diagnosis (76).

#### Similar mutations

A group of algorithms, Non-negative Matrix Factorization, and principal component analysis of 29 cancers revealed six amino acid mutation signatures with Glu > Lys and Arg > His mutations being the most notable characteristics of identified mutation signatures, while Sample-level analysis revealed that some cancers are heterogeneous, others are largely dominated by one type of mutation. Based on data from the P53 database, they found that the frequencies of p53 mutation in colorectal, head and neck, pancreatic, stomach, breast cancer, and liver are 43, 42, 34, 32, 22 and 31%, respectively (74).

## In diagnostic radiology

Four articles included in the review discussed the advancement of artificial intelligence and ML techniques in diagnostic radiology. Total kidney volume (TKV) and total liver volume (TLV) were measured in patients with autosomal dominant polycystic kidney disease (ADPKD) using a deep learning-based fully automated segmentation method (57), and a fully automated method for semantic segmentation of kidney cysts from MR images of patients with ADPKD was studied (51). Promising results were found in both studies, with the methods accurately detecting modifications in these parameters as correctly as manual tracing (interclass correlation coefficients, 0.998 and 0.996, respectively) with low bias and high precision (<0.1%2.7% for TKV and − 1.6%3.1% for TLV); this was comparable with inter-reader variability of manual tracing (<0.13.5% for TKV and − 1.5%4.8% for TLV), while the algorithm used in second study also accurately segmented renal cysts from kidney tissue without user intervention.

Successfully predicting which patients would succeed or fail 18 months after the start of behavioral weight loss treatment by using a higher-order SVD (HOSVD) in conjunction with machine learning applied to pre-treatment functional brain networks generated from functional magnetic resonance imaging (fMRI) yielded accurate results (>95% accuracy level). Those with BMIs below the median lost an average of 2.87 percent of their body weight (95% CI = 1.41 to 4.33) while those with BMIs above the median lost an average of 13.96 percent (95% CI = 11.86 to 16.05) (60). Unlike previous studies using only 1 CNN model, this study proposed a Computer Aided Diagnosis (CAD) system that used deep transfer learning to classify chest X-ray images into two classes: “Pneumonia” and “Normal.” The proposed system used an ensemble framework that took into account the decision scores derived from three Convolutional Neural Network (CNN) models to form a weighted average ensemble. These models were GoogLeNet, ResNet-18, and DenseNet-121. Using a five-fold cross-validation scheme, an evaluation of a single CNN model on two pneumonia chest X-ray datasets yielded an accuracy rate of 98.81%, sensitivity rate of 98.80%, precision rate of 98.82%, and f1-score of 98.79% on the Kermany dataset, and an accuracy rate of 86.86%, sensitivity rate of 87.02%, precision rate of 86.89% on the RSNA challenge dataset (75).

Using deep machine learning techniques, Dekhil et al. (63) created an automated autism diagnostic system, paving the way for tailored medication for autism. The stacked autoencoder was fed the power spectral densities (PSDs) of time courses that corresponded to the spatial activation zones in order to construct a classifier using probabilistic support vector machines. Our machine-learning method achieved 90% sensitivity, specificity, and accuracy, using data acquired from the National Database of Autism Research (63).

## In medical genetics and molecular pathology

Sepsis is a detrimental response of immunity against any infection of varying etiology. The detection of various prognostic and diagnostic biomarkers can aid in better diagnosis and treatment of septic patients. Around 170 biomarkers have been tested yet for use in sepsis. Schaack et al. (79) examined the predictive accuracy of Differential Expression (DE) analysis, Support Vector Machines (SVMs), Deep Neural Networks (DNNs), and other machine-learning approaches when applied to blood samples taken from patients with sepsis. In contrast to DE genes, which only demonstrated partial identification of samples (AUC >0.99 and > 0.96, respectively), deep learning approaches demonstrated outstanding diagnostic accuracy (79). Previous studies have demonstrated the existence of a spectrum of clinical phenotypes among septic patients, as well as a range of possible therapeutic approaches. Using a generalized estimating equation and k-means clustering to derive phenotypes, Kudo et al. (89) performed a secondary analysis of three multicenter registries to investigate the associations between thrombomodulin treatment and 28-day and in-hospital mortality for each phenotype of sepsis with coagulopathy. The derivation cohort consisted of 3,694 patients, and from them, they were able to identify four distinct sepsis phenotypes. Organ dysfunction, increased mortality, and elevated levels of FDP and D-dimer were all characteristics of cluster dA (*n* = 323) patients with severe coagulopathy. The cluster dB showed considerable coagulopathy with severe illness. Disease severity was intermediate or mild in cluster dC, and low in cluster dD, both with or without coagulopathy. Among patients with cluster dA, thrombomodulin was related to a decreased risk of death throughout the course of 28 days (adjusted risk difference [RD]: 17.8% [95% CI 28.7 to 6.9%]) and while in the hospital (adjusted RD: 17.7% [95% CI 27.6 to 7.9%]) (89).

Red blood cell (RBC) membrane fluidity is affected by multiple factors related to chronic hyperglycemia, oxidative stress, and metabolic alterations triggered by absolute insulin deficiency, rather than the glycosylation of a single protein a, and has shown varying alterations in its fluid variability in patients with Type 1 diabetes mellitus (T1DM). Maulucci et al. (81) conducted case–control research to further examine this. There was a total of 26 participants: 18 with type 1 diabetes and 8 healthy controls. T1DM patients were divided into two groups: those without problems and with a shorter illness duration (15 years; *n* = 11, group G1), and those with complications and a longer disease duration (>15 years; *n* = 7, group G2). Researchers employed functional two-photon microscopy to examine RBCs from type 1 diabetics and discovered fluidity maps at sub-micron scales, as well as a fluid split between fluid and stiff domains due to the effects of glycosylation and oxidation on membrane fluidity. Distinction patterns varied significantly between healthy individuals, people with G1, and those with G2. There was a statistically significant (p0.001) distinction between healthy, G1, and G2 patients, therefore a decision-support system (DSS) was built utilizing a machine learning technique to quantify the data and distinguish the fluidity patterns in RBCs. The primary drawback of this study was the small sample size and the possibility of bias in the examination of other environmental variables that may also affect membrane fluidity. Given that commercial image-based high-throughput systems can analyze multiwall plates offering computer-controlled, automated picture collection and processing. Once confirmed, this method showed the potential to be employed as a clinical test (81).

Topological data analysis (TDA), a machine learning technique used to discover natural sub-groups of Traumatic brain injury (TBI) patients, was recently studied by Nielson et al. (55), who found that it was able to identify a distinct diagnostic subgroup of patients with unfavorable outcome after mild TBI, whose outcomes were significantly predicted by the presence of specific genetic polymorphisms (SNP) in PARP1, which suggested that PARP1 may be a useful biomarker in estimating the patient trajectory in mild TBI patients. Extremely high rates of post-traumatic stress disorder (PTSD) and enrichment in the heterozygous allele of the PARP1 SNPs, which are associated with cellular responses to stress and DNA damage, were found in a large subpopulation of mild TBI subjects who recovered poorly and tended to deteriorate from 3 to 6 months after injury. Once the TBI syndromic space was established using the pre-selected CDEs, a full set of data for all patients was tackled to discover novel predictors of recovery after TBI, including several SNPs. This was accomplished by applying TDA to data from multiple CT and MR imaging and neuropsychological domains (55).

V66M mutation is the most common mutation in Brain-derived neurotrophic factor (BDNF) protein, damaging its structure and function and leading to many psychiatric disorders. In order to know the exact structural and functional effect of the mutation on BDNF protein, computational methodology was used via 9 algorithms for functional and structural prediction of the V66M mutation. PolyPhen-2 and SIFT predicted the V66M mutation to be deleterious, destabilizing by I-Mutant and Molecular dynamics (MD) analyses suggested that the mutation affects the essential motions, hydrogen-bonding, and secondary structure at pre- and pro-domain of BDNF essential for the protein to maintain its function and activity while its flexibility and surface-to-volume ratio remain unaffected (90).

Leucocyte epigenomic biomarkers were investigated in case–control research by Bahado-Singh et al. (65) to diagnose Alzheimer’s disease (AD) and provide light on its molecular pathophysiology. Twenty-four patients with late-onset Alzheimer’s disease (LOAD) and twenty-four controls had their DNA methylation profiles analyzed using the Infinium Methylation EPIC Bead Chip array. Using six different AI methods, researchers were able to identify 171 unique genes in AD patients with substantially (FDR p0.05) differently methylated intragenic CpGs. As a result of employing 283,143 intragenic and 244,246 intergenic/extragenic CpGs, all AI techniques were able to accurately predict AD (AUC 0.93). Genes such as CR1L and CTSV (abnormal cerebral cortex morphology), S1PR1 (central nervous system inflammation), and LTB4R were epigenetically modified (inflammatory response). Some of these genes have been connected to Alzheimer’s disease and other forms of dementia. Since there is evidence connecting poor cerebral blood flow, heart disease, and Alzheimer’s disease, it is intriguing to consider the role of the differentially methylated CTSV and PRMT5 (ventricular hypertrophy and dilation) genes (65).

Despite the enormous amount of information about gene-disease associations in the literature, the time-intensive curation procedure slows down the data extraction process. Bhasuran et al. (91) created an automatic extraction of gene-disease connections from the literature using joint ensemble learning to address this issue. After evaluation, the machine learning method delivered the expected results on the EUADR, GAD, CoMAGC, and PolySearch corpora of 85.34, 83.93, 87.39, and 85.57% F-measure, respectively. In comparison to earlier existing methodologies, these new results produced improved F-measures (91).

Research has been conducted on computer-aided polyp detection in gastric gastroscopy throughout the past few decades. The majority of prior techniques for diagnosing gastrointestinal polyps relied on the texture, color, location, and elliptical feature shapes alone or in combination. These characteristics were, however, typically created by hand. In this recent study, Zhang et al. (58) evaluated the use of an improved Single Shot MultiBox Detector (SSD) architecture known as SSD-GPNet for detecting gastric polyps, recognizing real-time detection with 50 frames per second (FPS) using Titan V, and improving the mean average precision (mAP) of detection. They put forth fresh pooling techniques that were deployed on the feature pyramid network to recover lost relevant data from Max-Pooling layers. Additionally, three new pooling levels known as Second Max-Pooling (Sec Max-Pooling), Second Min-Pooling (Sec Min-Pooling), and Min-Pooling were created to reduce the possibility of reusing the information lost from the Max-Pooling layers. The outcomes demonstrated that SSD-GPNet outperformed standard SSD in small-sized gastric polyps, improving the mAP by 2.1%, demonstrating that SSD-GPNet could extract and recognize more features from images (58). The model’s main flaw was that it was trained using SSD-GPNet and had more parameters than was necessary, which led to a modest decline in time performance.

Though research has connected the limbic system of the brain’s habenula to numerous mental disorders, including Major Depressive Disorder (MDD), it is too small and has a low contrast for the human eye to distinguish on radiological scans. For this, 7 Tesla MRI imaging in a Korean study used an automated segmentation and Habenula volume estimation method. The approach was built on a semantic segmentation network powered by deep learning. The DNNs strategy for Habenula volume estimation in 7 MRI was demonstrably applicable and had potential for usage in mental neuroimaging investigations due to its high dice similarity coefficient (0.852) and reproducibility (59).

In their clinical study, Lyra et al. (61) evaluated deep learning (DL)--based real-time vital sign extraction utilizing contactless infrared thermography on a dataset of 26 ICU patients (IRT). The dataset was applied to the object detectors YOLOv4 and YOLOv4-Tiny for training and validation. Head detection was used to quantify the body surface temperature (BST) trend, and chest movements were used to extract the respiratory rate (RR) using an optical flow (OF) method. A hold-out test dataset of 6 patients was used for validation, and the results showed good detector performance (0.75 intersections over union, 0.94 mean average Precision). The breathing rate was determined by an optical flow algorithm from the chest area. The trial produced encouraging findings for reliable label detection. The YOLOv4 model on the test dataset was found to have an IoU of 0.70, however, the small model displayed a superior IoU of 0.75. A mean absolute error of 2.69 bpm was found in the results (61).

The development of atherosclerotic heart disorders has been strongly linked to elevated levels of low-density lipoprotein cholesterol (LDL-C). It has been demonstrated that lowering LDL-C improves outcomes in both primary and secondary preventive populations. By comparing the method’s correlation to direct LDL-C with the Fried Ewald and Martin-Hopkins equations for LDL-C estimation, Singh et al (63) studied a machine learning (ML) approach using the RF model (the Weill Cornell model) to estimate Low-density lipoprotein cholesterol (LDL-C) from standard profiles. The Weill Cornell model had a correlation coefficient between estimated and measured LDL-C levels of 0.982, compared to 0.950 for Fried Ewald and 0.962 for the Martin-Hopkins approach. The TG > 500 and LDL-C 70 subgroups were stratified by LDL-C and TG values, and the Weill-Cornell model consistently performed better. The major limitation convenience sample of lipid profile data made at a solitary tertiary care facility in New York was used to build the Weill Cornell model. Although the Weill Cornell model had already undergone internal validation, external validation was still necessary to verify both the model’s generalizability and its accuracy across a variety of patient groups. The current analysis concentrated on creating and validating the model across a range of LDL-C and TG levels; however, it did not include patient-level analysis to ascertain the impact of specific clinical characteristics (such as ethnicity, the presence of kidney disease, the use of lipid-lowering drug therapies, etc.) on the performance of the model, opening the door for future research on the model (82).

Correct risk assessment and recommendation of the best treatment choices can be made with the aid of individuals with psychiatric diseases who exhibit positive valence symptoms. In their study, Dai et al. (83) tested the effectiveness of several CNN models in predicting the intensity of positive valence symptoms in patients with psychiatric illnesses based on initial records of psychiatric evaluations. The records included question-and-answer pairs and unstructured text, with the latter being tokenized and normalized. Convolutional and max pooling layers were used in various configurations to automatically learn significant characteristics from various word representations. The results showed that normalization of the semi-structured contents can increase the mean absolute error (MAE) among all CNN configurations to 0.785 and that the best CNN had an MAE of 0.539, supporting the hypothesis (83).

It can be difficult to diagnose glaucoma, particularly in the early stages. However, glaucoma can be treated early in order to prevent vision loss, if detected in the premature stages. Clinicians would benefit greatly from a machine-learning model that detects glaucoma more accurately. As a result, Kim et al. (66) evaluated four machine learning algorithms in their study to build a glaucoma prediction model that could interpret features from the measurement of retinal nerve fiber layer (RNFL) thickness and visual field (VF) to diagnose the condition. These algorithms were C5.0, random forest (RF), support vector machine (SVM), and k-nearest neighbor (KNN). With classification accuracy of 0.98, sensitivity of 0.983, specificity of 0.975, and AUC of 0.979, the RF model outperformed the C5.0, SVM, and KNN models in terms of performance. The research could be viewed as a breakthrough in the understanding of the main ocular illnesses and their therapies using machine intelligence and precision medicine (66).

As Black-box ML approaches do not cover the area of cause-effect relationships in detail, Rieg et al. (69) presented a white-box ML algorithm, which used a C5.0 model to categorize cardiovascular rhythms into four classes of (i) atrial fibrillation and atrial flutter, (ii) tachycardia (iii), sinus bradycardia and (iv) sinus rhythm based on five features (ventricular rate, RR-Interval variation, atrial rate, age and difference between longest and shortest RR-Interval) extracted from ECG data. The findings showed a generic tree structure that might provide each class with a different value to set it apart from ECG (69). One of this method’s drawbacks was that the model might become unstable if the database were modified. Additionally, linear relationships cannot be accurately represented by a decision tree (69).

The concept that the gene expression profile of a cell affected by a certain disease contains characteristic patterns linked to that disease and using these profiles to extract information for a better diagnosis and assessment was evaluated in a study by Gaudel et al. (70), who used multi-scale neural-network based framework that integrates gene expression data associated to diseases with gene–pathway information. Multiclass logistic regression (MLR), Random Forest (RF), Bernoulli Naive Bayes, and Support Vector Machine (SVM) algorithms were used to test the model’s classification performance, and they found that MLR and GPD performed better, or at least on par, with these competing methods when compared to RF, Naive Bayes (nB), and SVM classifiers, as measured by our three metrics. Given that MLR contained four times as many parameters than GPD, it might be said to be the most sophisticated neural network model. According to the study’s findings, employing biological knowledge to direct the layout of neural networks did not enhance classification performance when compared to MLR and only marginally improved it when compared to an RF classifier (70).

## Conclusion

In today’s world, AI has become a crucial tool in different fields. The future of AI in healthcare setup is also promising, especially in the case of extremely demanding fields like precision medicine. High heterogeneity and variability encountered in personalized medicine, particularly in domains like preventive medicine, diagnostic medicine, drug design, and treatment outcome can be tackled with different ML algorithms as they can automatically uncover complex non-linear relationships from heterogeneous sources of data, thus providing superior output in prediction tasks. However, the broader acceptance of AI in medicine is hampered because of the dearth of standardized protocols between many datasets producing biases in medical data, which, if used to design novel algorithms, might be less accurate and challenging to be generalizable. Furthermore, with highly personalized information being available publicly, data privacy can be an important issue. That is why, the investment of more time, money, and skills is imperative to deal with these shortcomings of AI applications in precision medicine for its widespread adoption in healthcare setup.

## Data availability statement

The original contributions presented in the study are included in the article/supplementary material, further inquiries can be directed to the corresponding authors.

## Author contributions

HM and MuS: conceptualization. HS, WM, MM, and MuS: writing – original draft. HM, MyS, SI, SJ, MK, and QM: review. MuS: final editing. All authors contributed to the article and approved the submitted version.

## Conflict of interest

The authors declare that the research was conducted in the absence of any commercial or financial relationships that could be construed as a potential conflict of interest.

## Publisher’s note

All claims expressed in this article are solely those of the authors and do not necessarily represent those of their affiliated organizations, or those of the publisher, the editors and the reviewers. Any product that may be evaluated in this article, or claim that may be made by its manufacturer, is not guaranteed or endorsed by the publisher.

## Figures and Tables

**Table:** 

BNs	Boolean Networks
CCLE	The Cancer Cell Line Encyclopedia
EHR	electronic health records
SVM	Support Vector Machine
ACI	acute cerebral ischemia
ECOS	Evolving connectionist system
DENFIS	Neuro-fuzzy inference system
tDCS	transcranial direct current stimulation
SVM	Support Vector Machine
CCLE	Cancer Cell Line Encyclopedia
ANN	Artificial Neural Network
Prisma	Preferred Reporting Items for Systematic Reviews and Meta-Analyses
DCNN	deep convolutional neural network
ML	machine learning
AI	Artificial intelligence
CDRP	Concatenated Diagnostic-Relapse Prognostic
KNN	k-nearest neighbor
RF	random forest
IPA	Ingenuity Pathway Analysis
LOAD	late-onset AD
SSD	Single Shot MultiBox Detector
SML	Supervised machine learning
TDA	Topological data analysis
GLM	Generalized Linear Model
LSTM	Long Short-Term Memory
RNN	Recurrent Neural Networks
TFI	Treatment Feature Interactions
CAMIL	Causal Modeling with Internal Layers
DPNN	Differential Prototypes Neural Network
GDSC	Genomics of Drug Sensitivity in Cancer
ANFIS	adaptive neuro-fuzzy inference
PAI	Personalized Advantage Index
ECOS	evolving connectionist systems
OCCT	oral carnitine challenge test
TMAO	trimethylamine N-oxide
TD	typically developing
DD	Developmental Disorders
ALS	Amyotrophic lateral sclerosis
GWAS	Genome-wide association studies
LR	logistic regression
MS	Multiple Sclerosis
VAR	Vector Autoregressive Model
AUC-ROC	Area under the Receiver Operating Characteristics
CAN	Care Assessment Needs
FA	food allergy
ECGs	electrocardiograms
cPSTA	Cardiac Phase Space Tomography Analysis
DSS	Decision-Support-System
GBM	gradient boosting machine
CART	classification, and regression trees
DT,	decision tree
GB	Gradient Boosting
MLP	Multilayer Perceptron
RFE	recursive feature elimination
RDoC	Research Domain Criteria
ICU	Intensive Care Unit
SUD	Substance Use Disorder
SL	Super-learning
EEG	electroencephalography
IPT	interpersonal psychotherapy
CT	cognitive therapy
CT	cortical thickness
MDD	major depressive disorder
SSRI	serotonin reuptake inhibitor
EMBARC	Establishing Moderators and Biosignatures of Antidepressant Response in Clinical Care
QRF	quantile regression forest
CCLE	Cell Line Encyclopaedia
T1DM	Type 1 diabetes mellitus
DE	Differential Expression
PSDs	power spectral densities
CNN	Convolutional Neural Network
CAD	Computer Aided Diagnosis
ADPKD	autosomal dominant polycystic kidney disease
MN	Melanocytic Nevus
ME	malignant Melanoma
SGD	Stochastic Gradient Descent
OS	Overall Survival
MCC	Matthews Correlation Coefficient
nSES	Neighbourhood socioeconomic
AML	acute myeloid leukemia
MRD	Minimal residual disease
NSCLC	non-small cell lung cancer
DCM	degenerative cervical myelopathy
GC	gastric cancer
BMI	body mass index
FEM	finite element models
RNFL	retinal nerve fiber layer
MAE	mean absolute error
LDL-C	low-density lipoprotein cholesterol
MDD	Major Depressive Disorder
AD	Alzheimer’s disease
BDNF	Brain-derived neurotrophic factor
PTSD	post-traumatic stress disorder
SNP	specific genetic polymorphisms
TBI	Traumatic brain injury

## References

[1] Steele AJ Denaxas SC Shah AD Hemingway H Luscombe NM. Machine learning models in electronic health records can outperform conventional survival models for predicting patient mortality in coronary artery disease. PLoS One. (2018) 13:e0202344. doi: 10.1371/journal.pone.0202344, PMID: 30169498 PMC6118376

[2] Weng SF Reps J Kai J Garibaldi JM Qureshi N. Can machine-learning improve cardiovascular risk prediction using routine clinical data? PLoS One. (2017) 12:e0174944. doi: 10.1371/journal.pone.0174944, PMID: 28376093 PMC5380334

[3] Stuckey TD Gammon RS Goswami R Depta JP Steuter JA Meine FJ III. Cardiac phase space tomography: a novel method of assessing coronary artery disease utilizing machine learning. PLoS One. (2018) 13:e0198603. doi: 10.1371/journal.pone.0198603, PMID: 30089110 PMC6082503

[4] Baydoun M Safatly L Abou Hassan OK Ghaziri H El Hajj A Isma'eel H. High precision digitization of paper-based ECG records: a step toward machine learning. IEEE J Translat Eng Health Med. (2019) 7:1–8. doi: 10.1109/JTEHM.2019.2949784, PMID: 32166049 PMC6876931

[5] Alag A. Machine learning approach yields epigenetic biomarkers of food allergy: a novel 13-gene signature to diagnose clinical reactivity. PLoS One. (2019) 14:e0218253. doi: 10.1371/journal.pone.0218253, PMID: 31216310 PMC6584060

[6] Parikh RB Linn KA Yan J Maciejewski ML Rosland A-M Volpp KG . A machine learning approach to identify distinct subgroups of veterans at risk for hospitalization or death using administrative and electronic health record data. PLoS One. (2021) 16:e0247203. doi: 10.1371/journal.pone.0247203, PMID: 33606819 PMC7894856

[7] Kim D. Predicting participation in Cancer screening programs with machine learning. arXiv preprint arXiv:2101.11614. (2021). doi: 10.48550/arXiv.2101.11614

[8] Haddaway NR Page MJ Pritchard CC McGuinness LA. PRISMA2020: an R package and Shiny app for producing PRISMA 2020-compliant flow diagrams, with interactivity for optimized digital transparency and. Open Synth Campbell Syst Rev. (2022) 18:e1230. doi: 10.1002/cl2.1230 PMC8958186 36911350

[9] Wu WK Panyod S Liu PY Chen CC Kao HL Chuang HL . Characterization of TMAO productivity from carnitine challenge facilitates personalized nutrition and microbiome signatures discovery. Microbiome. (2020) 8:162. doi: 10.1186/s40168-020-00912-y, PMID: 33213511 PMC7676756

[10] Abedi V Goyal N Tsivgoulis G Hosseinichimeh N Hontecillas R Bassaganya-Riera J . Novel screening tool for stroke using artificial neural network. Stroke. (2017) 48:1678–81. doi: 10.1161/STROKEAHA.117.017033, PMID: 28438906

[11] Brennan M Puri S Ozrazgat-Baslanti T Feng Z Ruppert M Hashemighouchani H . Comparing clinical judgment with the MySurgeryRisk algorithm for preoperative risk assessment: a pilot usability study. Surgery. (2019) 165:1035–45. doi: 10.1016/j.surg.2019.01.002, PMID: 30792011 PMC6502657

[12] 11Karpati T Leventer-Roberts M Feldman B Cohen-Stavi C Raz I Balicer R. Patient clusters based on HbA1c trajectories: a step toward individualized medicine in type 2 diabetes. PLoS One. (2018) 13:e0207096. doi: 10.1371/journal.pone.0207096, PMID: 30427908 PMC6235308

[13] Marshall MR Song Q Ma TM MacDonell SG Kasabov NK. Evolving connectionist system versus algebraic formulas for prediction of renal function from serum creatinine. Kidney Int. (2005) 67:1944–54. doi: 10.1111/j.1523-1755.2005.00293.x, PMID: 15840042

[14] Mencattini A Mosciano F Comes MC Di Gregorio T Raguso G Daprati E . An emotional modulation model as signature for the identification of children developmental disorders. Sci Rep. (2018) 8:14487. doi: 10.1038/s41598-018-32454-7, PMID: 30262838 PMC6160482

[15] Romero-Rosales B-L Tamez-Pena J-G Nicolini H Moreno-Treviño M-G Trevino V. Improving predictive models for Alzheimer’s disease using GWAS data by incorporating misclassified samples modeling. PLoS One. (2020) 15:e0232103. doi: 10.1371/journal.pone.0232103, PMID: 32324812 PMC7179850

[16] Saha S Pagnozzi A Bourgeat P George JM Bradford DK Colditz PB . Predicting motor outcome in preterm infants from very early brain diffusion MRI using a deep learning convolutional neural network (CNN) model. NeuroImage. (2020) 215:116807:116807. doi: 10.1016/j.neuroimage.2020.116807 32278897

[17] Lu F Zheng Y Cleveland H Burton C Madigan D. Bayesian hierarchical vector autoregressive models for patient-level predictive modeling. PLoS One. (2018) 13:e0208082. doi: 10.1371/journal.pone.0208082, PMID: 30550560 PMC6294362

[18] Popov P Bizin I Gromiha M Frishman D. Prediction of disease-associated mutations in the transmembrane regions of proteins with known 3D structure. PLoS One. (2019) 14:e0219452. doi: 10.1371/journal.pone.0219452, PMID: 31291347 PMC6620012

[19] Zhao Y Healy BC Rotstein D Guttmann CRG Bakshi R Weiner HL . Exploration of machine learning techniques in predicting multiple sclerosis disease course. PLoS One. (2017) 12:e0174866. doi: 10.1371/journal.pone.0174866, PMID: 28379999 PMC5381810

[20] Blasco H Patin F Descat A Garçon G Corcia P Gelé P . A pharmaco-metabolomics approach in a clinical trial of ALS: identification of predictive markers of progression. PLoS One. (2018) 13:e0198116. doi: 10.1371/journal.pone.0198116, PMID: 29870556 PMC5988280

[21] Ott PA Hu Z Keskin DB Shukla SA Sun J Bozym DJ . An immunogenic personal neoantigen vaccine for patients with melanoma. Nature. (2017) 547:217–21. doi: 10.1038/nature22991 28678778 PMC5577644

[22] Li Q Shi R Liang F. Drug sensitivity prediction with high-dimensional mixture regression. PLoS One. (2019) 14:e0212108. doi: 10.1371/journal.pone.0212108, PMID: 30811440 PMC6392252

[23] Miranda SP Baião FA Fleck JL Piccolo SR. Predicting drug sensitivity of cancer cells based on DNA methylation levels. PLoS One. (2021) 16:e0238757. doi: 10.1371/journal.pone.0238757, PMID: 34506489 PMC8432830

[24] Fang Y Xu P Yang J Qin Y. A quantile regression forest-based method to predict drug response and assess prediction reliability. PLoS One. (2018) 13:e0205155. doi: 10.1371/journal.pone.0205155, PMID: 30289891 PMC6173405

[25] Vitali F Cohen LD Demartini A Amato A Eterno V Zambelli A . A network-based data integration approach to support drug repurposing and multi-target therapies in triple negative breast cancer. PLoS One. (2016) 11:e0162407. doi: 10.1371/journal.pone.0162407, PMID: 27632168 PMC5025072

[26] Tang Z Zhang T Yang B Su J Song Q. spaCI: deciphering spatial cellular communications through adaptive graph model. Brief Bioinform. (2023) 24:bbac563. doi: 10.1093/bib/bbac563, PMID: 36545790 PMC9851335

[27] Bartlett EA DeLorenzo C Sharma P Yang J Zhang M Petkova E . Pretreatment and early-treatment cortical thickness is associated with SSRI treatment response in major depressive disorder. Neuropsychopharmacology. (2018) 43:2221–30. doi: 10.1038/s41386-018-0122-9, PMID: 29955151 PMC6135779

[28] Albizu A Fang R Indahlastari A O'Shea A Stolte SE See KB . Machine learning and individual variability in electric field characteristics predict tDCS treatment response. Brain Stimul. (2020) 13:1753–64. doi: 10.1016/j.brs.2020.10.001, PMID: 33049412 PMC7731513

[29] Nguyen CT Luckett DJ Kahkoska AR Shearrer GE Spruijt-Metz D Davis JN . Estimating individualized treatment regimes from crossover designs. Biometrics. (2020) 76:778–88. doi: 10.1111/biom.13186, PMID: 31743424 PMC7234899

[30] Rajpurkar P Yang J Dass N Vale V Keller AS Irvin J . Evaluation of a machine learning model based on pretreatment symptoms and electroencephalographic features to predict outcomes of antidepressant treatment in adults with depression: a prespecified secondary analysis of a randomized clinical trial. JAMA Netw Open. (2020) 3:e206653. doi: 10.1001/jamanetworkopen.2020.6653, PMID: 32568399 PMC7309440

[31] Tomalin LE Kim J Correa da Rosa J Lee J Fitz LJ Berstein G . Early quantification of systemic inflammatory proteins predicts long-term treatment response to tofacitinib and etanercept. J Invest Dermatol. (2020) 140:1026–34. doi: 10.1016/j.jid.2019.09.023, PMID: 31705874

[32] Webb CA Trivedi MH Cohen ZD Dillon DG Fournier JC Goer F . Personalized prediction of antidepressant v. placebo response: evidence from the EMBARC study. Psychol Med. (2019) 49:1118–27. doi: 10.1017/S0033291718001708, PMID: 29962359 PMC6314923

[33] Kazemipoor M Hajifaraji M Radzi CW Shamshirband S Petković D Mat Kiah ML. Appraisal of adaptive neuro-fuzzy computing technique for estimating anti-obesity properties of a medicinal plant. Comput Methods Prog Biomed. (2015) 118:69–76. doi: 10.1016/j.cmpb.2014.10.006 25453384

[34] Yoon HJ Kang J Park H Sohn I Lee S-H Lee HY. Deciphering the tumor microenvironment through radiomics in non-small cell lung cancer: correlation with immune profiles. PLoS One. (2020) 15:e0231227. doi: 10.1371/journal.pone.0231227, PMID: 32251447 PMC7135211

[35] Bradley A Van der Meer R McKay CJ. A prognostic Bayesian network that makes personalized predictions of poor prognostic outcome post resection of pancreatic ductal adenocarcinoma. PLoS One. (2019) 14:e0222270. doi: 10.1371/journal.pone.0222270, PMID: 31498836 PMC6733484

[36] Huang C Mezencev R McDonald JF Vannberg F. Open-source machine-learning algorithms for the prediction of optimal cancer drug therapies. PLoS One. (2017) 12:e0186906. doi: 10.1371/journal.pone.0186906 29073279 PMC5658085

[37] Lind AP Anderson PC. Predicting drug activity against cancer cells by random forest models based on minimal genomic information and chemical properties. PLoS One. (2019) 14:e0219774. doi: 10.1371/journal.pone.0219774, PMID: 31295321 PMC6622537

[38] Kaissis G Ziegelmayer S Lohöfer F Steiger K Algül H Muckenhuber A . A machine learning algorithm predicts molecular subtypes in pancreatic ductal adenocarcinoma with differential response to gemcitabine-based versus FOLFIRINOX chemotherapy. PLoS One. (2019) 14:e0218642. doi: 10.1371/journal.pone.0218642, PMID: 31577805 PMC6774515

[39] Kleinerman A Rosenfeld A Benrimoh D Fratila R Armstrong C Mehltretter J . Treatment selection using prototyping in latent-space with application to depression treatment. PLoS One. (2021) 16:e0258400. doi: 10.1371/journal.pone.0258400, PMID: 34767577 PMC8589171

[40] Mirchi N Bissonnette V Yilmaz R Ledwos N Winkler-Schwartz A Del Maestro RF. The virtual operative assistant: an explainable artificial intelligence tool for simulation-based training in surgery and medicine. PLoS One. (2020) 15:e0229596. doi: 10.1371/journal.pone.0229596, PMID: 32106247 PMC7046231

[41] Christie SA Conroy AS Callcut RA Hubbard AE Cohen MJ. Dynamic multi-outcome prediction after injury: applying adaptive machine learning for precision medicine in trauma. PLoS One. (2019) 14:e0213836. doi: 10.1371/journal.pone.0213836, PMID: 30970030 PMC6457612

[42] Ramella S Fiore M Greco C Cordelli E Sicilia R Merone M . A radiomic approach for adaptive radiotherapy in non-small cell lung cancer patients. PLoS One. (2018) 13:e0207455. doi: 10.1371/journal.pone.0207455, PMID: 30462705 PMC6248970

[43] Sharma T Parihar J Singh S. Intelligent chatbot for prediction and management of stress In: 2021 11th international conference on cloud computing, data science & engineering (confluence). Piscataway: IEEE (2021). 937–41.

[44] Sheikhalishahi S Balaraman V Osmani V. Benchmarking machine learning models on multi-Centre eICU critical care dataset. PLoS One. (2020) 15:e0235424. doi: 10.1371/journal.pone.0235424, PMID: 32614874 PMC7332047

[45] McCoy TH Jr Pellegrini AM Perlis RH. Differences among research domain criteria score trajectories by diagnostic and statistical manual categorical diagnosis during inpatient hospitalization. PLoS One. (2020) 15:e0237698. doi: 10.1371/journal.pone.0237698, PMID: 32842139 PMC7447552

[46] Jiang X Wells A Brufsky A Neapolitan R. A clinical decision support system learned from data to personalize treatment recommendations towards preventing breast cancer metastasis. PLoS One. (2019) 14:e0213292. doi: 10.1371/journal.pone.0213292, PMID: 30849111 PMC6407919

[47] Lin YW Zhou Y Faghri F Shaw MJ Campbell RH. Analysis and prediction of unplanned intensive care unit readmission using recurrent neural networks with long short-term memory. PLoS One. (2019) 14:e0218942. doi: 10.1371/journal.pone.0218942, PMID: 31283759 PMC6613707

[48] Merali ZG Witiw CD Badhiwala JH Wilson JR Fehlings MG. Using a machine learning approach to predict outcome after surgery for degenerative cervical myelopathy. PLoS One. (2019) 14:e0215133. doi: 10.1371/journal.pone.0215133, PMID: 30947300 PMC6448910

[49] Jiang Z Cinti C Taranta M Mattioli E Schena E Singh S . Network assessment of demethylation treatment in melanoma: differential transcriptome-methylome and antigen profile signatures. PLoS One. (2018) 13:e0206686. doi: 10.1371/journal.pone.0206686, PMID: 30485296 PMC6261551

[50] Panda S. Use of robots in dentistry: a fact or a fiction. Indian J Forensic Med Toxicol. (2020) 14

[51] Kline TL Edwards ME Fetzer J Gregory AV Anaam D Metzger AJ . Automatic semantic segmentation of kidney cysts in MR images of patients affected by autosomal-dominant polycystic kidney disease. Abdom Radiol. (2021) 46:1053–61. doi: 10.1007/s00261-020-02748-4, PMID: 32940759 PMC7940295

[52] Wang D Xu J Zhang Z Li S Zhang X Zhou Y . Evaluation of rectal cancer circumferential resection margin using faster region-based convolutional neural network in high-resolution magnetic resonance images. Dis Colon Rectum. (2020) 63:143–51. doi: 10.1097/DCR.0000000000001519, PMID: 31842158

[53] Gates E Lin JS Weinberg JS Prabhu SS Hamilton J Hazle JD . Imaging-based algorithm for the local grading of glioma. AJNR Am J Neuroradiol. (2020) 41:400–7. doi: 10.3174/ajnr.A6405, PMID: 32029466 PMC7077885

[54] Wang XX Ding Y Wang SW Dong D Li HL Chen J . Intratumoral and peritumoral radiomics analysis for preoperative Lauren classification in gastric cancer. Cancer Imaging. (2020) 20:83. doi: 10.1186/s40644-020-00358-3, PMID: 33228815 PMC7684959

[55] Nielson JL Cooper SR Yue JK Sorani MD Inoue T Yuh EL . Uncovering precision phenotype-biomarker associations in traumatic brain injury using topological data analysis. PLoS One. (2017) 12:e0169490. doi: 10.1371/journal.pone.0169490, PMID: 28257413 PMC5336356

[56] Ko BS Wang YF Li JL Li CC Weng PF Hsu SC . Clinically validated machine learning algorithm for detecting residual diseases with multicolor flow cytometry analysis in acute myeloid leukemia and myelodysplastic syndrome. EBioMedicine. (2018) 37:91–100. doi: 10.1016/j.ebiom.2018.10.042 30361063 PMC6284584

[57] van Gastel M Edwards ME Torres VE Erickson BJ Gansevoort RT Kline TL. Automatic measurement of kidney and liver volumes from MR images of patients affected by autosomal dominant polycystic kidney disease. J Am Soc Nephrol. (2019) 30:1514–22. doi: 10.1681/ASN.2018090902, PMID: 31270136 PMC6683702

[58] Zhang X Chen F Yu T An J Huang Z Liu J . Real-time gastric polyp detection using convolutional neural networks. PLoS One. (2019) 14:e0214133. doi: 10.1371/journal.pone.0214133, PMID: 30908513 PMC6433439

[59] Lim SH Yoon J Kim YJ Kang CK Cho SE Kim KG . Reproducibility of automated habenula segmentation via deep learning in major depressive disorder and normal controls with 7 tesla MRI. Sci Rep. (2021) 11:13445. doi: 10.1038/s41598-021-92952-z, PMID: 34188141 PMC8241874

[60] Mokhtari F Rejeski WJ Zhu Y Wu G Simpson SL Burdette JH . Dynamic fMRI networks predict success in a behavioral weight loss program among older adults. NeuroImage. (2018) 173:421–33. doi: 10.1016/j.neuroimage.2018.02.025, PMID: 29471100 PMC5911254

[61] Lyra S Mayer L Ou L Chen D Timms P Tay A . A deep learning-based camera approach for vital sign monitoring using thermography images for ICU patients. Sensors. (2021) 21:1495. doi: 10.3390/s21041495, PMID: 33670066 PMC7926634

[62] de Jong EEC van Elmpt W Rizzo S Colarieti A Spitaleri G Leijenaar RTH . Applicability of a prognostic CT-based radiomic signature model trained on stage I-III non-small cell lung cancer in stage IV non-small cell lung cancer. Lung Cancer. (2018) 124:6–11. doi: 10.1016/j.lungcan.2018.07.023, PMID: 30268481

[63] Dekhil O Hajjdiab H Shalaby A Ali MT Ayinde B Switala A . Using resting state functional MRI to build a personalized autism diagnosis system. PLoS One. (2018) 13:e0206351. doi: 10.1371/journal.pone.0206351 30379950 PMC6209234

[64] Badgujar NV Tarapara BV Shah FD. Computational analysis of high-risk SNPs in human CHK2 gene responsible for hereditary breast cancer: a functional and structural impact. PLoS One. (2019) 14:e0220711. doi: 10.1371/journal.pone.0220711, PMID: 31398194 PMC6688789

[65] Bahado-Singh RO Vishweswaraiah S Aydas B Yilmaz A Metpally RP Carey DJ . Artificial intelligence and leukocyte epigenomics: evaluation and prediction of late-onset Alzheimer’s disease. PLoS One. (2021) 16:e0248375. doi: 10.1371/journal.pone.0248375, PMID: 33788842 PMC8011726

[66] Kim SJ Cho KJ Oh S. Development of machine learning models for diagnosis of glaucoma. PLoS One. (2017) 12:e0177726. doi: 10.1371/journal.pone.0177726, PMID: 28542342 PMC5441603

[67] Lynch SM Handorf E Sorice KA Blackman E Bealin L Giri VN . The effect of neighborhood social environment on prostate cancer development in black and white men at high risk for prostate cancer. PLoS One. (2020) 15:e0237332. doi: 10.1371/journal.pone.0237332 32790761 PMC7425919

[68] Keek S Sanduleanu S Wesseling F de Roest R van den Brekel M van der Heijden M . Computed tomography-derived radiomic signature of head and neck squamous cell carcinoma (peri)tumoral tissue for the prediction of locoregional recurrence and distant metastasis after concurrent chemo-radiotherapy. PLoS One. (2020) 15:e0232639. doi: 10.1371/journal.pone.0232639, PMID: 32442178 PMC7244120

[69] Rieg T Frick J Baumgartl H Buettner R. Demonstration of the potential of white-box machine learning approaches to gain insights from cardiovascular disease electrocardiograms. PLoS One. (2020) 15:e0243615. doi: 10.1371/journal.pone.0243615 33332440 PMC7746264

[70] Gaudelet T Malod-Dognin N Sánchez-Valle J Pancaldi V Valencia A Pržulj N. Unveiling new disease, pathway, and gene associations via multi-scale neural network. PLoS One. (2020) 15:e0231059. doi: 10.1371/journal.pone.0231059, PMID: 32251458 PMC7135208

[71] Maggio V Chierici M Jurman G Furlanello C. Distillation of the clinical algorithm improves prognosis by multi-task deep learning in high-risk neuroblastoma. PLoS One. (2018) 13:e0208924. doi: 10.1371/journal.pone.0208924, PMID: 30532223 PMC6285384

[72] Takahashi Y Sone K Noda K Yoshida K Toyohara Y Kato K . Automated system for diagnosing endometrial cancer by adopting deep-learning technology in hysteroscopy. PLoS One. (2021) 16:e0248526. doi: 10.1371/journal.pone.0248526, PMID: 33788887 PMC8011803

[73] Jiang Y Chen L Zhang H Xiao X. Breast cancer histopathological image classification using convolutional neural networks with small SE-ResNet module. PLoS One. (2019) 14:e0214587. doi: 10.1371/journal.pone.0214587, PMID: 30925170 PMC6440620

[74] Szpiech ZA Strauli NB White KA Ruiz DG Jacobson MP Barber DL . Prominent features of the amino acid mutation landscape in cancer. PLoS One. (2017) 12:e0183273. doi: 10.1371/journal.pone.0183273, PMID: 28837668 PMC5570307

[75] Kundu R Das R Geem ZW Han G-T Sarkar R. Pneumonia detection in chest X-ray images using an ensemble of deep learning models. PLoS One. (2021) 16:e0256630. doi: 10.1371/journal.pone.0256630, PMID: 34492046 PMC8423280

[76] Araújo DC Veloso AA de Oliveira Filho RS Giraud MN Raniero LJ Ferreira LM . Finding reduced Raman spectroscopy fingerprint of skin samples for melanoma diagnosis through machine learning. Artif Intell Med. (2021) 120:102161. doi: 10.1016/j.artmed.2021.102161 34629149

[77] Goetz CM Arnetz JE Sudan S Arnetz BB. Perceptions of virtual primary care physicians: a focus group study of medical and data science graduate students. PLoS One. (2020) 15:e0243641. doi: 10.1371/journal.pone.0243641, PMID: 33332409 PMC7745971

[78] Guijo-Rubio D Briceño J Gutiérrez PA Ayllón MD Ciria R Hervás-Martínez C. Statistical methods versus machine learning techniques for donor-recipient matching in liver transplantation. PLoS One. (2021) 16:e0252068. doi: 10.1371/journal.pone.0252068, PMID: 34019601 PMC8139468

[79] Schaack D Weigand MA Uhle F. Comparison of machine-learning methodologies for accurate diagnosis of sepsis using microarray gene expression data. PLoS One. (2021) 16:e0251800. doi: 10.1371/journal.pone.0251800, PMID: 33999966 PMC8128240

[80] Gennatas ED Wu A Braunstein SE Morin O Chen WC Magill ST . Preoperative and postoperative prediction of long-term meningioma outcomes. PLoS One. (2018) 13:e0204161. doi: 10.1371/journal.pone.0204161 30235308 PMC6147484

[81] Maulucci G Cordelli E Rizzi A De Leva F Papi M Ciasca G . Phase separation of the plasma membrane in human red blood cells as a potential tool for diagnosis and progression monitoring of type 1 diabetes mellitus. PLoS One. (2017) 12:e0184109. doi: 10.1371/journal.pone.0184109, PMID: 28880900 PMC5589169

[82] Singh G Hussain Y Xu Z Sholle E Michalak K Dolan K . Comparing a novel machine learning method to the Friedewald formula and Martin-Hopkins equation for low-density lipoprotein estimation. PLoS One. (2020) 15:e0239934. doi: 10.1371/journal.pone.0239934, PMID: 32997716 PMC7526877

[83] Dai H-J Jonnagaddala J. Assessing the severity of positive valence symptoms in initial psychiatric evaluation records: should we use convolutional neural networks? PLoS One. (2018) 13:e0204493. doi: 10.1371/journal.pone.0204493, PMID: 30325934 PMC6191093

[84] Freidlin B Simon R. Adaptive signature design: an adaptive clinical trial design for generating and prospectively testing a gene expression signature for sensitive patients. Clin Cancer Res. (2005) 11:7872–8. doi: 10.1158/1078-0432.CCR-05-0605 16278411

[85] Dreno B Thompson JF Smithers BM Santinami M Jouary T Gutzmer R . MAGE-A3 immunotherapeutic as adjuvant therapy for patients with resected, MAGE-A3-positive, stage III melanoma (DERMA): a double-blind, randomised, placebo-controlled, phase 3 trial. Lancet Oncol. (2018) 19:916–29. doi: 10.1016/S1470-2045(18)30254-7 29908991

[86] van Bronswijk SC DeRubeis RJ Lemmens L Peeters F Keefe JR Cohen ZD . Precision medicine for long-term depression outcomes using the personalized advantage index approach: cognitive therapy or interpersonal psychotherapy? Psychol Med. (2021) 51:279–89. doi: 10.1017/S0033291719003192, PMID: 31753043 PMC7893512

[87] Maciukiewicz M Marshe VS Hauschild AC Foster JA Rotzinger S Kennedy JL . GWAS-based machine learning approach to predict duloxetine response in major depressive disorder. J Psychiatr Res. (2018) 99:62–8. doi: 10.1016/j.jpsychires.2017.12.009, PMID: 29407288

[88] Acion L Kelmansky D van der Laan M Sahker E Jones D Arndt S. Use of a machine learning framework to predict substance use disorder treatment success. PLoS One. (2017) 12:e0175383. doi: 10.1371/journal.pone.0175383, PMID: 28394905 PMC5386258

[89] Kudo D Goto T Uchimido R Hayakawa M Yamakawa K Abe T . Coagulation phenotypes in sepsis and effects of recombinant human thrombomodulin: an analysis of three multicentre observational studies. Crit Care. (2021) 25:1–11. 33741010 10.1186/s13054-021-03541-5 PMC7978458

[90] De Oliveira CCS Pereira GRC De Alcantara JYS Antunes D Caffarena ER De Mesquita JF. In silico analysis of the V66M variant of human BDNF in psychiatric disorders: an approach to precision medicine. PLoS One. (2019) 14:e0215508. doi: 10.1371/journal.pone.0215508, PMID: 30998730 PMC6472887

[91] Bhasuran B Natarajan J. Automatic extraction of gene-disease associations from literature using joint ensemble learning. PLoS One. (2018) 13:e0200699. doi: 10.1371/journal.pone.0200699, PMID: 30048465 PMC6061985

